# Combining callers improves the detection of copy number variants from whole-genome sequencing

**DOI:** 10.1038/s41431-021-00983-x

**Published:** 2021-11-08

**Authors:** Marie Coutelier, Manuel Holtgrewe, Marten Jäger, Ricarda Flöttman, Martin A. Mensah, Malte Spielmann, Peter Krawitz, Denise Horn, Dieter Beule, Stefan Mundlos

**Affiliations:** 1 Institute of Medical and Human Genetics, Charité Universitätsmedizin, Berlin, Germany; 2 Core Unit Bioinformatics, Berlin Institute of Health, Berlin, Germany; 3 Charité Universitätsmedizin Berlin, Berlin, Germany; 4 Core Unit Genomics, Berlin Institute of Health, Berlin, Germany; 5 Berlin Institute of Health (BIH), Anna-Louisa-Karsch-Str. 2, 10178 Berlin, Germany; 6 Max Planck Institute for Molecular Genetics, Berlin, Germany; 7 Institut für Genomische Statistik und Bioinformatik, Bonn, Germany; 8 Max Delbrück Center for Molecular Medicine, Berlin, Germany; 9 Present Address: Institute of Human Genetics, University of Lübeck, Lübeck, Germany

**Keywords:** DNA sequencing, Genome informatics

## Abstract

Copy Number Variants (CNVs) are deletions, duplications or insertions larger than 50 base pairs. They account for a large percentage of the normal genome variation and play major roles in human pathology. While array-based approaches have long been used to detect them in clinical practice, whole-genome sequencing (WGS) bears the promise to allow concomitant exploration of CNVs and smaller variants. However, accurately calling CNVs from WGS remains a difficult computational task, for which a consensus is still lacking. In this paper, we explore practical calling options to reach the best compromise between sensitivity and sensibility. We show that callers based on different signal (paired-end reads, split reads, coverage depth) yield complementary results. We suggest approaches combining four selected callers (Manta, Delly, ERDS, CNVnator) and a regenotyping tool (SV2), and show that this is applicable in everyday practice in terms of computation time and further interpretation. We demonstrate the superiority of these approaches over array-based Comparative Genomic Hybridization (aCGH), specifically regarding the lack of resolution in breakpoint definition and the detection of potentially relevant CNVs. Finally, we confirm our results on the NA12878 benchmark genome, as well as one clinically validated sample. In conclusion, we suggest that WGS constitutes a timely and economically valid alternative to the combination of aCGH and whole-exome sequencing.

## Introduction

Structural variations (SVs) are DNA variations larger than 50 base pairs (bp) [1–3] and include copy number variants (CNVs) (deletions, duplications and insertions), and copy number neutral variants (inversions and translocations). SVs are considered responsible for 50–95% of human samples sequence difference to the reference genome [3, 4]. They are prominent in human diseases, with 15% of patients with intellectual disability or schizophrenia harboring clinically relevant CNVs [5, 6]. They can alter the sequence of dosage-sensitive genes, lead to the expression of fusion transcripts or modify the regulatory landscape of a gene [7, 8], by altering the three-dimensional organization of genomes in topologically associated domains [9], which can for example lead to enhancer adoption [7].

The techniques developed for SVs detection evolved towards higher throughput and better resolution. Karyotyping allows to detect the larger scale ones, such as trisomy 21 [10], or t(9;22) translocation leading to BCR/ABL fusion transcript expression [11]. Molecular karyotyping relies on the simultaneous hybridization of two differentially labeled DNA samples (test and control) to an array with oligonucleotide probes and encompasses both high-resolution microarray-based Comparative Genomic Hybridization (aCGH) and single nucleotide polymorphism (SNP) arrays. They reach a theoretical resolution of 1–3 kilobases (kb) with commercially available 1M arrays [12]. Whole-exome sequencing (WES) allows genome-wide identification of disease-causing coding single nucleotide variants (SNVs) and small insertion-deletions, but has limited abilities to detect larger SVs [13].

Whole-genome sequencing (WGS) allows to analyze non-coding regions and to detect both balanced and unbalanced SVs at an unprecedented resolution. It outdoes WES for smaller variants detection [14] and aCGH for CNV calling [15]. While SV calling from short-read WGS remains challenging [16], combining tools might improve the results [17]. Algorithms indeed rely on several signal types: discordant read pairs (with abnormal distance or orientation), split-reads, depth of coverage, or local read assembly. Callers using one or more of these approaches exhibit different calls size ranges, breakpoint precision and false discovery rates [18] and suffer from considerable lack of reproducibility [19].

In this work, we use 24 patients with congenital limb malformations to explore the relevance of several computational tools aiming at CNV detection from WGS. We suggest different approaches, applicable in everyday practice, bringing more resolution to the call breakpoints than aCGH, and detecting a higher, but manageable, amount of calls. We suggest that WGS could be used as a first-line single test to detect a variety of variants.

## Materials and methods

### Subjects, ethics approval and aCGH

DNA was prepared from blood using standard procedures. All individuals provided written informed consent to participate in the study, approved by the Charité Universitätsmedizin Berlin ethics committee. aCGH was performed according to standard procedures [20]. Detailed methods are available in Supplementary Fig. S1.

### Whole-genome sequencing

Whole-genome sequencing was performed for the probands and their parents to allow compared visual examination, but data from the index case only was used for CNV calling. Libraries were prepared with the TruSeq DNA PCR Free (350) library kit and sequenced on HiSeq X (Macrogen, Korea). Raw images and base calls were generated through the integrated analysis software RTA2 (Real Time Analysis 2). Conversion of the BCL binary to FASTQ was performed with the Illumina package bcl2fastq2-v2.20.2, with demultiplexing option set to default and without trimming the adapters. Reads were aligned to the hg19 reference sequence with BWA-MEM v0.7.12q. SNVs were called with the GATK [1] HaplotypeCaller, v3.7-0-gcfedb67. Between 614,880,099 and 1,027,077,956 reads were produced per patient (average 803,031,274), with 95.46–99.8% of mapped reads. Mean coverage ranged between 27.6× and 43× (average 34×), with 40.5–88% of the reference covered at least 30× (average 67.7%). The bam file for individual NA12878 was downloaded from the Genome in a Bottle github page (https://github.com/genome-in-a-bottle/giab_data_indexes). Reference SVs sets were downloaded from the phase 3 of the 1000 Genomes Project (ftp://ftp.1000genomes.ebi.ac.uk/vol1/ftp/phase3/integrated_sv_map/).

### CNV calling from WGS

CNV calling was performed with coverage-based callers: CNVnator [21] (v0.3.3, default options, bin size 100), ERDS [22] (v1.1, default parameters), FREEC [23] (default hg19_len100bp mappability file, breakpoint threshold at 0.1, window value of 1000) and cnvkit [24] (v0.9.1a0, default -m threshold parameters and mappability options); callers using paired-end reads and split-reads: Delly2 [25] (v0.7.1, with the cohort re-genotyping option, merged with bcftools v1.7) and Manta [26] (v1.2.1, default parameters); and a mixed caller: Vaquita [27] (v0.4.0, default parameters). For the 3430 and NA12878 samples, Delly v0.7.6, Manta v1.6.0, CNVnator v0.4.1 and ERDS v1.1 were used via Miniconda3. Manta calls for sample NA12878 were issued from the original publication [26]. Calls were re-genotyped with SV2 [28], a support-vector machine-based software estimating SV genotype likelihoods (v1.4.0, with the -M option); filtered with SnpSift [29] (v4.2) for non-reference status in the index case using the GNU parallel tool [30]; and compared with the bedtools [31] suite tool intersect (v2.27.2). They were considered shared when their reciprocal overlap was above 50%.

### CNV calls visual classification

**Fig. 1:**
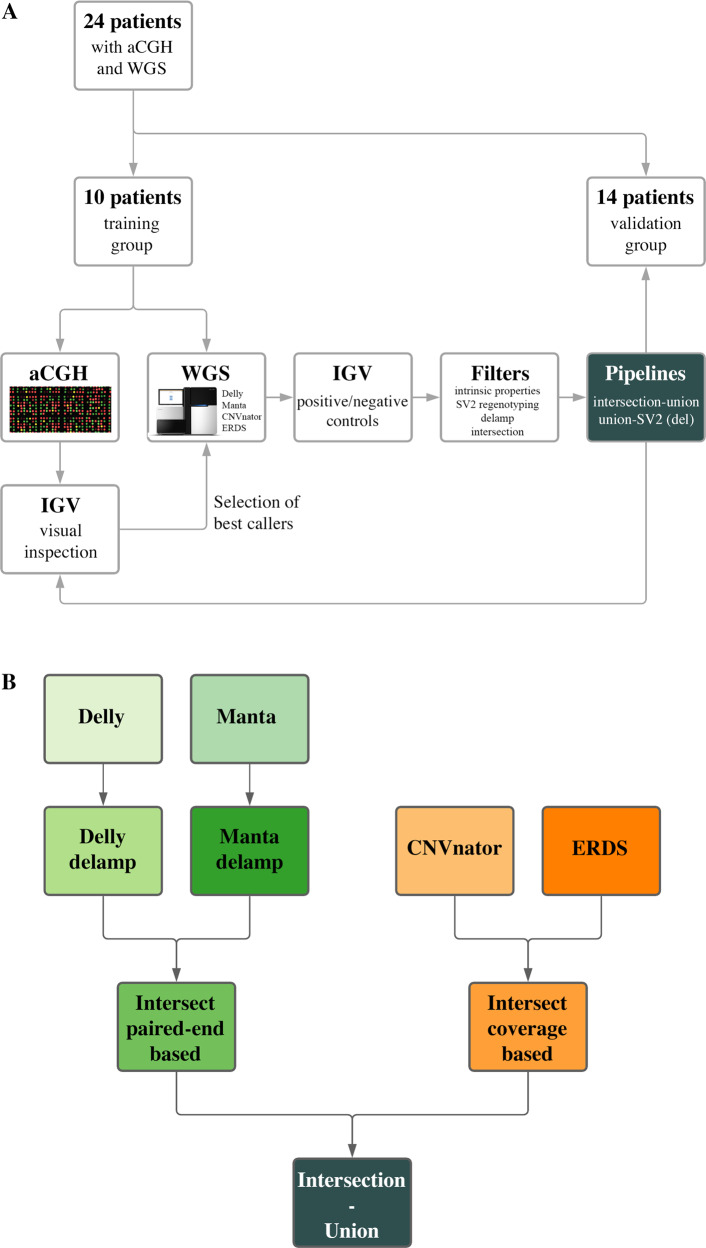
Schematics of the paper approach and one suggested pipeline. **A** From 24 patients with limb malformations, 10 were randomly selected as a training group. Their aCGH calls were visually inspected to select four callers best able to detect them from WGS data. Calls from these callers were in turn inspected to constitute sets of true positive and false positive calls, as to test filtering and combined calling options. Those options were then validated on the fourteen remaining patients. **B** The intersection-union approach is suggested for both deletions and gains. The calls from with Delly and Manta (paired-end based callers) are first filtered for calls matching a call from the opposite type (“delamp”). Calls from each pair of callers supported by the same signal are then intersected with 75% reciprocal overlap. The calls are finally joined to form the final calls set. The whole process takes less than 12 h on a cluster node with Intel(R) Xeon(R) CPU E5-2650 v2 @ 2.60 GHz (32 threads in total) with 126 GB Ram.

Ten patients from the cohort of 24 were randomly selected as the training group (Fig. 1). For a random sampling of 2026 calls from 4 WGS callers (Delly, Manta, ERDS, CNVnator), the alignments of reads were examined and compared to those of the proband’s parents in the Integrative Genomics Viewer [32] (IGV, v2.3.90). True positive calls were supported by the presence of a coverage drop, paired-end abnormal signal or split-reads (Supplementary Fig. S2). Shared calls showed similar profiles in the index and both parents (gain, homozygous deletion, or heterozygous deletion in all) and can either match shared true positive calls, or alignment artifacts. False positive calls were not confirmed by the visualization in IGV, while no conclusion could be made for the calls labeled doubtful. True positive and false positive calls were used as ground truth sets to test the filtering options. aCGH calls included one additional category, opposite calls (Supplementary Fig. S3).

### Filtering and combined strategies to improve WGS calling

Ground truth sets were used to compute performance statistics for each WGS caller, without or with further filtering of the calls. Filtering options included: re-genotyping with SV2, threshold on paired-end and split-read support fraction (Delly, at least one of the latter above 0.3), threshold on the adjusted *p* value (CNVnator, <0.5), exclusion of calls simultaneously labeled as gain and deletion (with 75% reciprocal overlap), and overlap with a call from another caller using the same signal (Delly/Manta; ERDS/CNVnator, at 50% and 75% reciprocal overlap). Strategies combining those callers and filtering options included: join the calls from the four callers; intersect the calls from caller pairs using the same approach, then combine both pools (“intersection-union” approach); and both of the latter approaches followed by SV2 re-genotyping.

### Calls intersection to known genome regions

WGS calls were further characterization with known genomic tracks: the SVs from the gnomAD database (SV v2.1, https://gnomad.broadinstitute.org/downloads#v2-structural-variants); the Repeat Masker reference, the DAC Blacklisted Regions and the Duke Excluded Regions (UCSC Table Browser, hg19); and reference alternated loci [33], lifted over to hg19.

### qPCR

qPCR was performed as described previously [34]. Three amplicons inside the CNV, one to the left, one to the right, and one on chromosome X were used. Primers are available on request.

## Results

### The landscape of CNV calls detected by each caller is extremely variable

**Fig. 2:**
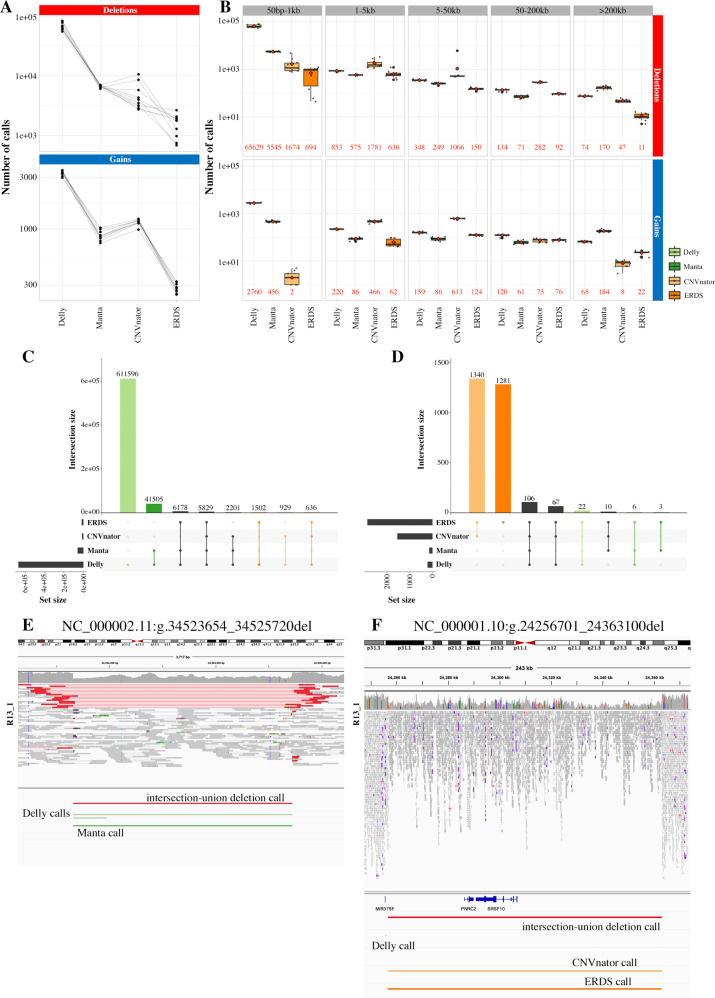
Description of the CNV calls landscape for four callers. **A** Total number of calls per patient in the training group for Delly, Manta, CNVnator and ERDS, per CNV type. The paired-end based callers, especially Delly, showed higher numbers of calls than the coverage-based ones, in particular ERDS. **B** Total number of calls per patient in the training group for the four callers, per CNV type and size range. The average counts are indicated in red. **C** Distribution of the overlaps of Delly deletions calls with the other callers. Most calls were unique to Delly (light green) or common to Manta only (darker green). **D** Distribution of the overlaps of ERDS gains calls with the other callers. Most calls were common to CNVnator only (light orange) or unique to ERDS (darker orange). All overlaps configurations are reported in Supplementary Fig. S5. **E** Example of deletion detected by both paired-end based callers, hence by the intersection-union approach, but not by the coverage-based callers. **F** Example of deletion detected by both coverage-based callers only, hence by the intersection-union approach. This call was shared in the trio and could be an alignment artifact.

Four callers were selected based on their ability to detect aCGH calls (Supplementary Fig. S4). The amount and range of detected calls varied a lot and the sets poorly overlapped (Fig. 2A–D, Supplementary Tables S1 and S2). The paired-end based callers detected more calls per patient and were especially enriched in small deletions. Coverage-based caller CNVnator detected significantly more deletions and gains in the 1–50 kb range. Callers using the same signal type showed higher overlap (Supplementary Table S2, Supplementary Fig. S5). Delly and CNVnator uniquely called more events than Manta and ERDS, whose calls were confirmed by another caller in around half the cases.

### The fraction of visually confirmed calls varies depending on call size and type

**Fig. 3:**
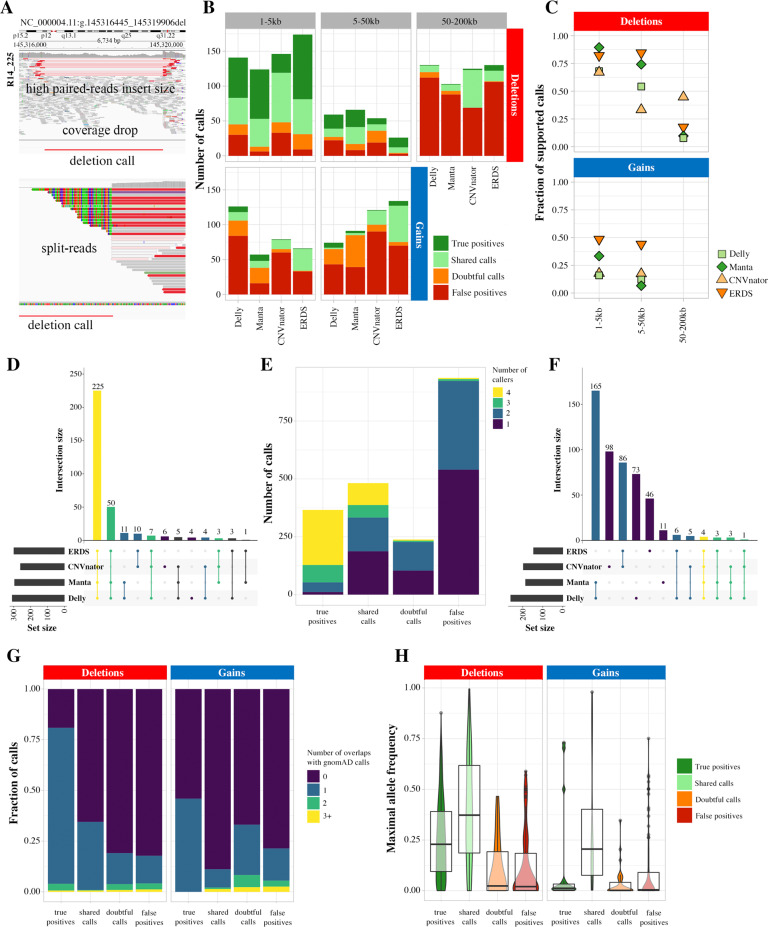
Visual inspection of WGS CNV calls. **A** Several elements allowed to establish the trueness of a call: a drop in coverage, paired-reads with abnormal insert size, or the presence of split-reads. Further examples are shown in Supplementary Fig. S2. **B** Repartition of visual inspection labels across 1278 deletions and 748 gains, per size range and caller. The large calls, as the gains, were less reliable. **C** Fraction of supported calls (true positive and shared calls) per size range and caller. ERDS specifically, and Manta, were generally more conservative than Delly and CNVnator. **D** Repartition of callers overlap for calls labeled as true positives. They were most frequently detected by four callers, or at least a pair of same-signal callers. **E** Number of callers simultaneously detecting calls with various inspection labels. The true positive calls were most frequently confirmed by an orthogonal caller. **F** Repartition of callers overlap for calls labeled as false positives. They were most frequently detected by a single caller, or at most a pair of same-signal callers. Number of overlaps (**G**) and maximal allele frequency of overlaps (**H**) of calls, per inspection label. True positive calls intersected more frequently with a gnomAD call, however with lower frequency than shared calls. Of note, false positive calls do not show particular recurrence in the database.

The visual inspection of a random selection of 1278 deletions and 748 gains established fractions of supported calls, based on true positive and shared calls, ranging from 6.6 to 89.5% (Fig. 3A–C, Supplementary Table S3). Small deletions were more reliable than gains or larger events. 1–50 kb deletions called by ERDS and Manta were the most reliable, and these two callers generally were more accurate. For Delly and CNVnator, true positive rates for deletions ranging from 1 to 5 kb were still above 50%. ERDS showed the highest supported fraction of large deletions and gains; calls however were often shared by the index case and the parents, hence could match alignment artifacts. Gains above 50 kb were almost never visually supported and came in vast majority from coverage-based callers (19/21). They were hence not included in the variants sets to avoid bias. True positive calls were often detected by all four callers; almost all of them were called by at least a pair of same-signal callers (Fig. 3D–F, Supplementary Fig. S6). False positive calls were often unique, or less frequently, unique to one pair of callers with shared signal. True positive calls intersected more frequently with calls from the gnomAD database (Fig. 3G, H), with lower maximal allele frequency than shared calls. False positive calls showed more overlap with regions matching alternating reference scaffolds (Supplementary Fig. S7) but not poor mappability or Repeat Masker elements.

### Filters improve the positive predictive value of WGS calls

**Fig. 4:**
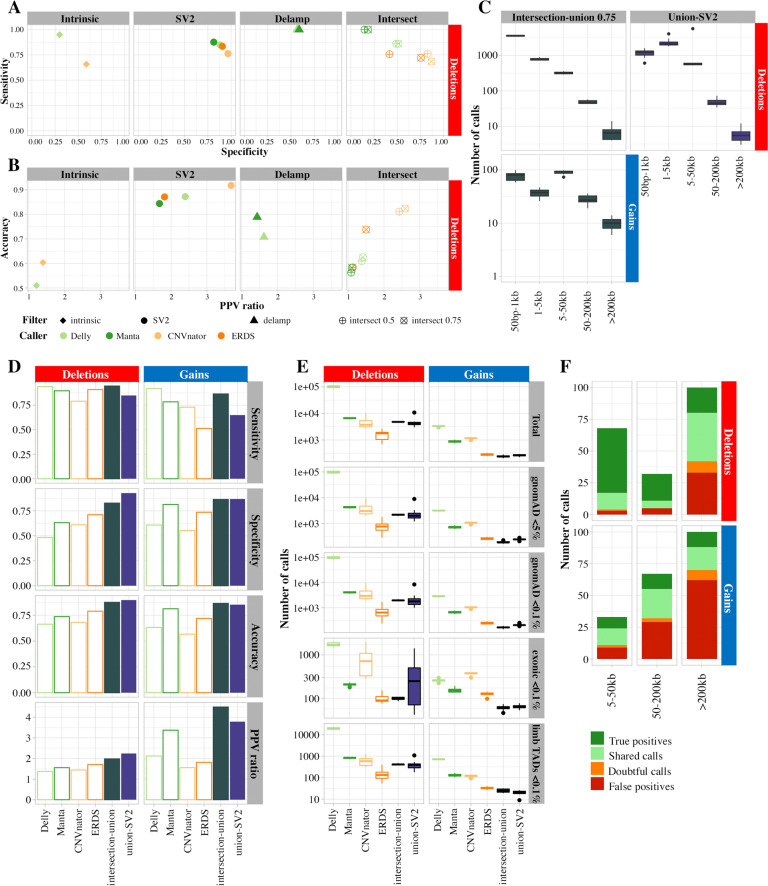
Performance of filters and pipelines on call sets accuracies. **A**, **B** Different filters, described in the method section, were tested on the call sets. Contingency values are reported for deletions here: sensitivity, specificity (**A**), accuracy, and ratio in predictive positive value (**B**). Filters on intrinsic calls properties show low specificities and accuracies. SV2 regenotyping performs well for deletions, with a 1.68–3.68 increase in positive predictive value. Delamp filters are not very specific, but really sensitive. Intersection performs better for coverage-based than paired-reads based callers. **C** Number of calls detected by each proposed approach. **D** Contingency values for single callers, versus intersection-union and union-SV2 approaches. The sensitivity of the calling does not increase much, if at all, by combining tools, but the specifity, the accuracy and the positive predictive ratio are markedly improved. **E** Quantification of calls yielded by single callers and the suggested approaches, after filtration for frequency, and regions of interest. **F** Repartition of visual inspection labels across 200 deletions and 200 gains issued from the intersection-union approach, per size range and caller. The supported fraction is significantly higher than for individual callers (Fig. 3).

The true (329 deletions, 37 gains) and false (505 deletions, 435 gains) positive calls were used to assess the performance of several filtering approaches described in the Methods (Supplementary Table S3). The estimated sensibilities, specificities, positive predictive values and accuracies based on these datasets varied depending on the callers and type of calls (Fig. 4AB, Supplementary Fig. S8, Supplementary Table S4). Filters on intrinsic properties showed low sensitivities and/or accuracies. The “delamp” filter was only applicable to paired-end callers, specifically for gains (Supplementary Fig. S9) and showed almost perfect sensitivity. The reciprocal overlap threshold used for the intersection filter did not affect its sensitivity but improved its specificity. SV2 regenotyping performed well for deletions but showed low sensitivities for gains.

### Combining tools allows a superior detection of CNV calls, within manageable limits

The combined positive and negative calls allowed to compare the estimated performance of single callers; paired-end callers with the “delamp” filter; and combined options described in the Methods (Supplementary Table S5, Supplementary Fig. S10). The intersection-union approach brought good results for both deletions and gains (Fig. 4C). The union approach followed by SV2 performed well for deletions, with lower sensitivity but higher specificity. Both approaches yielded calls in all size ranges, including large gains (Supplementary Table S6, Fig. 4B). Further inspection of 200 deletions established visually true positive fractions ranging from 12 to 75% (Supplementary Table S7, Fig. 4F), markedly improved compared to unique callers. The reliability of the calls decreases when the size range increase and calls above 200 kb are almost never visually confirmed. Four gains and eleven deletions from the intersection-union approach were assessed by qPCR (Supplementary Table S8). Two deletions were not confirmed; they were recurrent in the cohort and overlapped with gnomAD calls with allele frequencies above 5% (Supplementary Fig. S11). A large proportion of calls were in aCGH targeted regions (Supplementary Table S9), and some included several probes (Supplementary Fig. S12). The number of calls yielded was in the lower end of the distribution for unique callers (Fig. 4D, Supplementary Table S10). Filtering for calls detected in more than 5% of alleles in gnomAD yielded, on average, 2180 deletions and 188 gains per patient. More stringency on the threshold, up to 0.1%, had limited additional effect. Among these extremely rare calls, 99 deletions and 61 gains intersected with exonic regions, and 414 deletions and 25 gains with topologically associated domains linked to limb malformation phenotypes.

### WGS increases CNV detection and breakpoint accuracy compared to aCGH

All aCGH calls of the training cohort were visually inspected (Supplementary Tables S11 and S12) and the overall validity of these labels was confirmed by intersecting gnomAD database calls (Supplementary Table S13, Supplementary Fig. S3). Most deletions (223/251) and gains (38/39) could be detected by at least one caller with a reciprocal overlap of 50% (Supplementary Fig. S4, Supplementary Table S14). Coverage-based callers performed better, possibly because they rely on similar signal as aCGH. Almost all missed calls matched a CNV call with lower overlap, due to the poor precision of aCGH regarding breakpoints, inherent to the sparse localization of probes (Supplementary Fig. S1). For the validation cohort, aCGH calls undetected by the intersection-union approach were visually inspected in IGV (Supplementary Table S15). A majority of the calls were false positives (48.6% of deletions, 91.9% of gains) or more precisely characterized by WGS (35.9%, 2.4%).

### The strategies proposed work on clinically relevant and benchmarked samples

Patient 3430, not included in the initial cohort, presented with mirror-image polydactyly of the hands and feet and a complex variant with two overlapping duplications at chr7q36.1and a breakpoint fusion of intron 1 of *SHH* and intron 8 of *KDM4C* [35]. The gain, initially classified as VUS, was correctly detected by aCGH, as well as by the intersection-union approach, being called by both coverage-based callers (Supplementary Fig. S13). WGS however was needed to elucidate the fusion between chromosomes, detected by paired-end callers, and to suggest dysregulation of *SHH* expression following formation of an *SHH-KDM4C* neo-TAD. Finally, we tested the approach on the NA12878 reference sample. Overall, the sensitivity is comparable to the best callers, even if lowered in some cases (Supplementary Table S16). However, the positive predictive value is most often increased, as the set of yielded calls is smaller than for all callers but ERDS. The callers used alone show marked variations in performance for different type and sizes of calls; while the intersection-union approach is consistently good (Supplementary Table S17).

## Discussion

CNVs play a major role in human genetic diversity and diseases [7, 36]. WGS outperforms aCGH to detect them [15] and WES for the calling of coding variants [14, 37], but remains underused as a first-line tool, due to the absence of consensus for SV calling. Most studies rely on high quality data (around 80x coverage [37]), optimization of the whole WGS pipeline from library preparation [2], use of a high number of different platforms [18], or design of new callers [27], which is not always applicable in practice. In this paper, we assessed, in a cohort of 24 patients, how CNV calling can be performed efficiently with currently available tools, from “standard” Illumina WGS with 30x average coverage. Our study relies in great part on visual inspection of the calls; however, qPCR confirmed that, while not perfect, this provides a good basis for detection performance estimation. We hence suggest that combining tools proves to be an efficient and applicable approach [17, 38].

Indeed, while the landscape of CNVs detected by each caller is highly variable, their specificities can be leveraged to obtain a more comprehensive call set. While callers based on similar signals show higher overlap, some, like Manta and ERDS, inherently have higher specificity. ERDS considers single-nucleotide variants zygosity [22], which could explain its higher accuracy. Additional filters improve the quality of CNV calls sets: removing the calls flagged as both deletions and gains, which is a specific pitfall of paired-end based callers; and using regenotyping tools, which strongly increases specificity while also decreasing specificity. We suggest combinations of tools that yield a good compromise between specificity and sensitivity, in a variety of CNVs types and sizes. Our pipeline also allowed to better detect the gains, and, to a certain extent, larger calls, notoriously less reliable. The detection performance however decreases with the size range of the calls.

The reader’s specific question will ultimately guide their computational choice and the acceptable trade-off between sensitivity and specificity. If their interest is in a small region with high biological presumption, a higher false positive rate might be tolerated in order to increase variants detection and juxtaposing several callers could be the method of choice. If they want to assess CNVs genome-wide, a higher predictive positive value would be required and the use of a regenotyping tool or the intersection of the calls would prove instrumental. In all cases, visual inspection of the calls, while imperfect, remains invaluable.

Calling is just the first step of an analysis workflow, and the need for comprehensive databases and accurate tools for annotation is strong, as exemplified by the several hundreds of rare calls we obtained. We show that filtering with higher stringency on the frequency of gnomAD calls does not exert a big effect on the set size. GnomAD v3 includes a reference of 433,371 SVs called from 14,891 genomes [38], which is instrumental but way below the small variants counterpart of the database. While WGS algorithms do not rely on comparison to a control set, the use of cohort information allows interpretation, but also improves the specificity of the calling [25, 39, 40], by accounting for local imbalances of coverage linked to GC content, or ubiquitous paired-end signal anomalies in repetitive regions. Functional annotation of the genome is still a limiting factor in the calls’ interpretation, and the prioritization of clinically relevant CNVs will heavily rely on better understanding of the non-coding genome. The fact that we could not identify the causative variant in our initial cohort of 24 patients is illustrative of the need for powerful tools to interpret them.

Calling CNVs from WGS hence remains challenging but outperforms aCGH. It detects significantly more relevant CNVs, in aCGH target size range and regions. The sparsity of aCGH probes limits the breakpoint localization accuracy, which is a strong drawback since overlapping SVs are not always linked to the same phenotype [41]. Breakpoint localization is accurate to the nucleotide with paired-end based callers and hindered by bin size for coverage-based algorithms; the latter has an optimum between 30 and 100 bp for CNVnator [21]. WGS also localizes the gains insertion site, which is crucial for variant interpretation, as exemplified by our patient with a *SHH-KDM4C* neo-TAD [35], or a duplication in *TENM3* explaining intellectual deficiency upon disruption of *IQSEC2* sequence [13, 37]. Both cases were detected, but unexplained, by aCGH. Finally, while this work focused on CNVs, WGS allows detecting balanced SVs and more complex events.

Just as aCGH, short-read sequencing has intrinsic limitations that can only be overcome by other sequencing or calling approaches. De novo assembly, locally or genome-wide, might allow removing some artifacts and detecting insertion of novel sequences [42], as shown in 150 Danish genomes [43]. Sequencing techniques allowing to span over short and long tandem repeats, such as long read sequencing or mate-pair sequencing, lead to the identification of numerous SVs including inversions, complex variants, and long tracks of repeats [44], but have high rates of false positive SNVs. Techniques gathering longer-range and/or haplotype-phased information such as 10x Genomics linked-reads [45], strand-specific sequencing [46], or HiC data [47], as well as combinations of multiple approaches [18] are efficient, but not yet applicable for a large number of patients in a reasonable monetary and time frame.

In conclusion, we show that WGS is a valid first-line option for CNV calling, as also suggested by other studies [2, 15]. We suggest combining tools relying on various signal types to increase CNV calling detection from short-read Illumina WGS, specifically regarding estimated predictive positive values. Annotation of the data is still limited but will be improved with more widespread use of WGS. Turn-around time and price are crucial criteria in the selection of a diagnosis method. Using multiple techniques increases both, hence we advocate that WGS, while not yet perfect, should be considered.

## Supplementary information

Supplemental Material

## Data Availability

Genetic data generated and/or analyzed during the current study (pseudonymized, grouped where possible, and minimized) are available from the corresponding authorupon reasonable request.
