# Supplementary material for: Combining callers improves the detection of copy number variants from whole-genome sequencing
Source: Eur J Hum Genet. 2021 Nov 8;30(2):178–86. doi: 10.1038/s41431-021-00983-x (PMC8821561; doi:10.1038/s41431-021-00983-x)
Supplement: Supplementary file 1 — Supplemental Material [file 41431_2021_983_MOESM1_ESM.pdf]

Supplementary Figure S1
aCGH CNV calling in the Charité Universitätsmedizin Berlin cohort

In 463 patients referred to the Charité Universitätsmedizin Berlin hospital, aCGH was performed with high resolution whole-genome (1M) oligonucleotide array (Agilent), following the manufacturer's recommendations. Analysis was performed with Feature Extraction v9.5.3.1 and Cytogenomics v4.0.3.12 softwares (Agilent), with the Default Analysis Method CGH v2 including an absolute log ratio threshold at 0.25.
A. Density distribution of the 962,354 distances between probes of the Agilent whole-genome 1M oligonucleotide array in automosomes and X-Y chromosomes, represented as base-10 logarithms. The majority of probes is separated by 1-10kb, which accounts for the resolution of the array. The mean distance between two probes is 3,121bp. B. Distribution of the number of calls per patient in the entire cohort, the 24 patients with limb malformations described in this study, and the ten patients selected as a test group. Deletions are colored in red, gains in blue. 24 to 67 deletions and 5 to 29 gains were called per patient, which was representative of the cohort of 463 patients analyzed in the institute. C. Practical resolution achieved in the test group. Deletions ranged from 1.3kb to 2.2Mb, with a mean of 62.9kb and a median of 12.2kb. Gains ranged from 1.3kb to 2.8Mb, with a mean of 130.1kb and a median of 29.6kb.

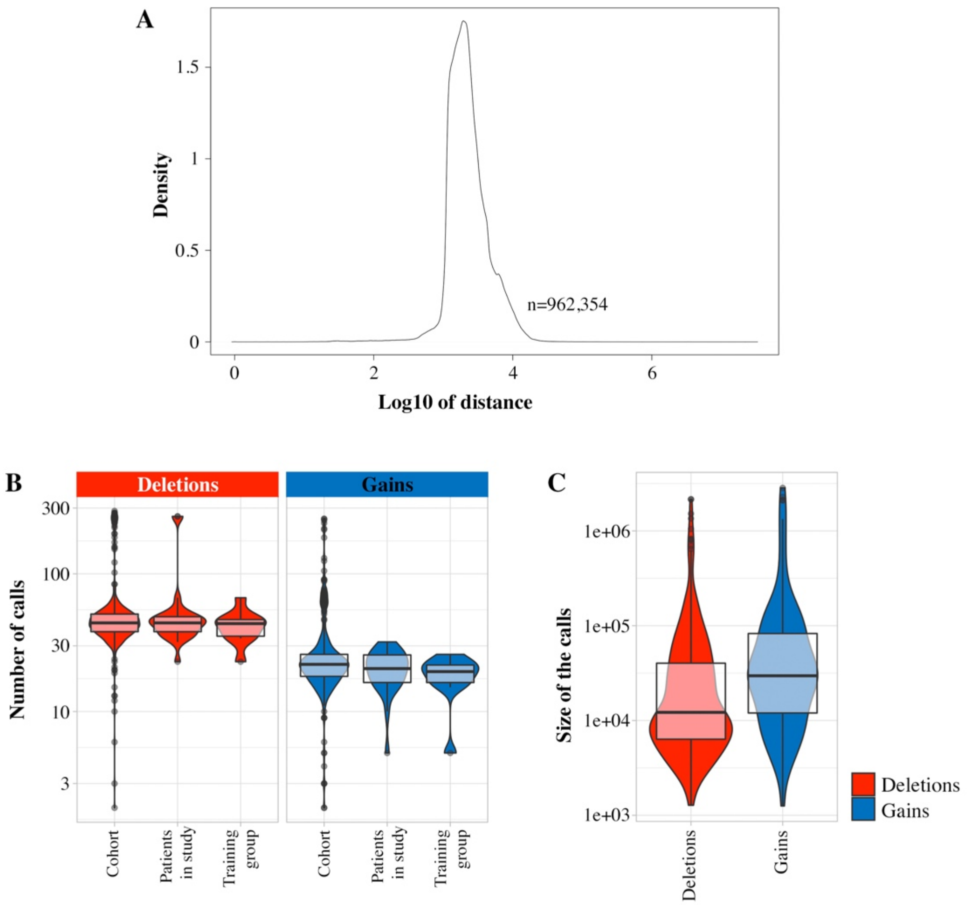

Supplementary Figure S2
Inspection evidence in WGS calls

A. Example of a true deletion call, objectified by lowered coverage in a heterozygous patient, absence of coverage in a homozygous patient, abnormal paired-reads insert sizes, and split reads. B. The same elements allow to confirm gain calls: increase in coverage depth, abnormal read pairs that appear sometimes inverted, and split reads. C. This example of false positive call shows an accumulation of reads whose mate maps to other chromosomes, in a region flagged by Dac mappability track (regions of the human genome with anomalous, unstructured, high read counts). D. Example of a false positive gain call. The region seems to match an alternative scaffold locus, with a complex call that is present at the heterozygous or homozygous state in various patients. E. Extreme example of accumulation of discordant paired-reads. This locus includes the HLA genes. Another patient showed a normal signal in the same region.

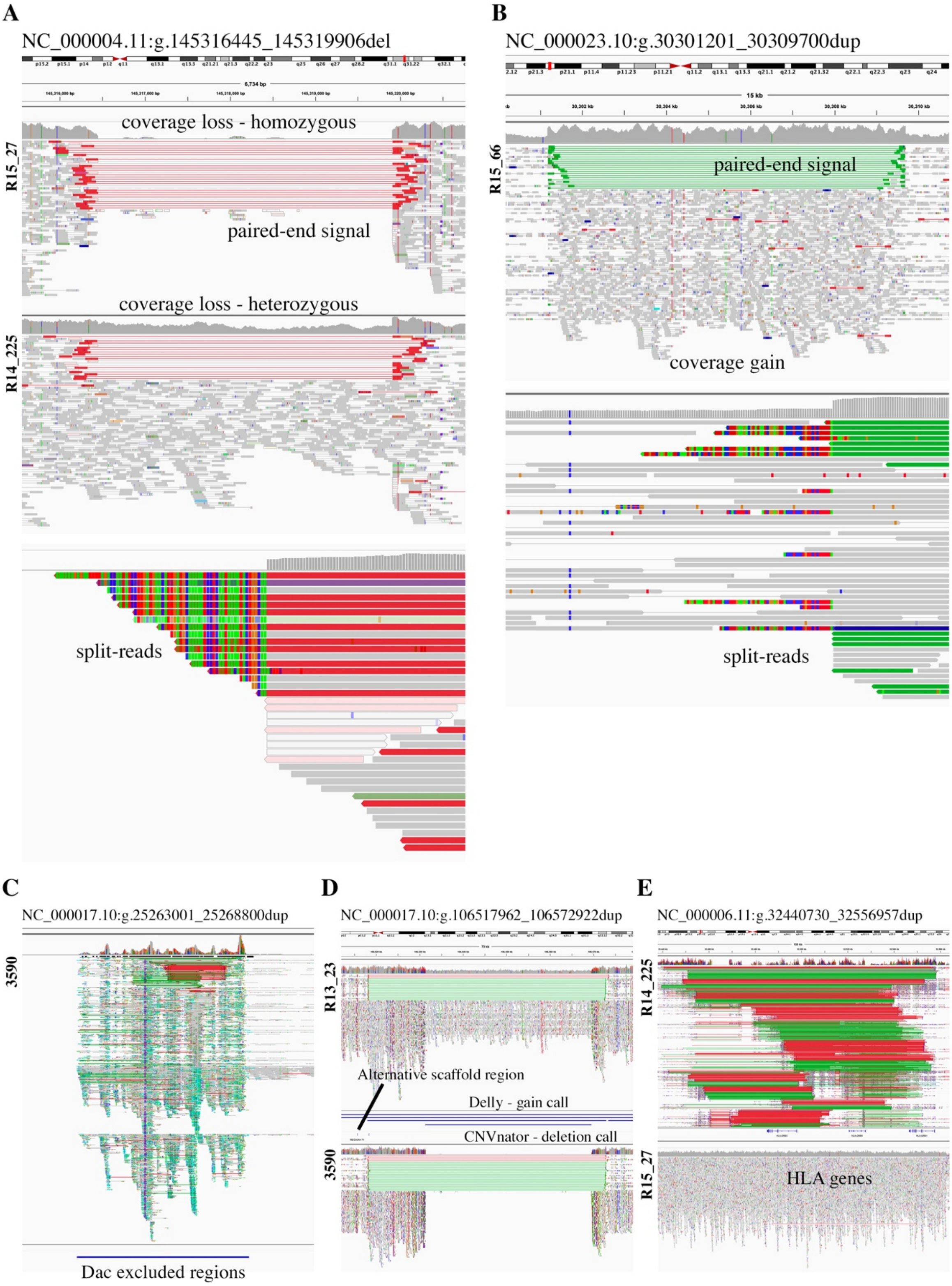

A
NC_000004.11:g.145316445_145319906del
coverage loss - homozygous
R15_27
paired-end signal
coverage loss - heterozygous
R14_225
split-reads
B
NC_000023.10:g.30301201_30309700dup
R15_66
paired-end signal
coverage gain
split-reads
C
NC_000017.10:g.25263001_25268800dup
3590
Dac excluded regions
D
NC_000017.10:g.106517962_106572922dup
R13_23
Alternative scaffold region
Delly - gain call
CNVnator - deletion call
3590
E
NC_000006.11:g.32440730_32556957dup
R14_225
R15_27
HLA genes

Supplementary Figure S3
Visual inspection of calls in aCGH

A. Visual inspection labels have been described in the Methods section and include true positive, shared, doubtful and false positive calls. For aCGH however, an additional category is needed: opposite calls. Those calls are a specific type of false positive, with flat coverage profile in the index at the locus, and a visually apparent deletion in the parent. This suggests they might be linked to frequent polymorphisms, also happening in the reference pool. Because aCGH is a comparative technique, the coverage would then appear higher in the index than in the controls, and a gain would be called. B. Repartition of inspection results for aCGH in the ten patients selected for the test cohort. C. Repartition of allele frequency of gnomAD overlapping SVs, for identical type of calls. aCGH calls were intersected with the reported SVs in the gnomAD database, assuming a reciprocal similarity of 50%. The distribution of the maximal allele frequency of the overlapping calls is reported, per category. Calls shared with at least one parent, as well as false positive calls, show overlap with known SVs with higher allele frequency. D. Repartition of allele frequency of gnomAD overlapping SVs, for opposite type of calls, e.g. deletions for gains. aCGH deletions were intersected with the reported gains in the gnomAD database, assuming a reciprocal similarity of 50%, and vice-versa. The distribution of the maximal allele frequency of the overlapping calls is reported, per category. Gains classified as opposite, for which a matching deletion was observed in IGV, often intersect with frequent deletions, which explain why they were called by aCGH in the first place. False positive calls also show frequent overlapping opposite SVs. E. Repartition of the sizes of the aCGH calls per inspected label. Supported deletions are on the lower range, while gains show similar profiles. F. Repartition of the log2ratio of the calls per inspected category. There is no profile difference between true and false positives. G. Repartition of the amounts of probes per call per inspected category. For deletions, false positives tend to have higher probes counts.

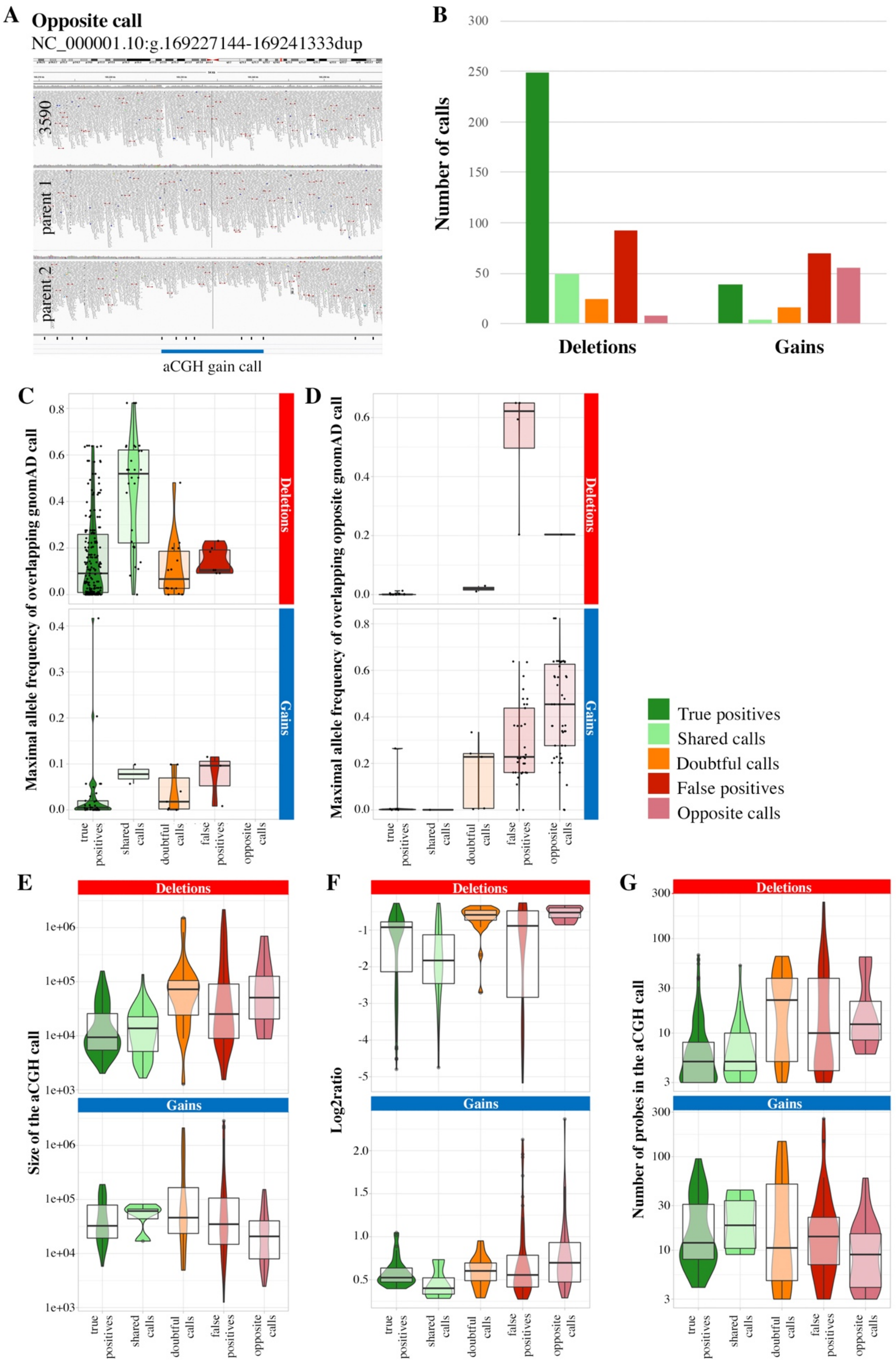
A
Opposite call
NC_000001.10:g.169227144-169241333dup
3590
parent 1
parent 2
aCGH gain call
B
Number of calls
Deletions
Gains
C
Maximal allele frequency of overlapping gnomAD call
D
Maximal allele frequency of overlapping opposite gnomAD call
Deletions
Gains
true positives
shared calls
doubtful calls
false positives
opposite calls
True positives
Shared calls
Doubtful calls
False positives
Opposite calls
E
Size of the aCGH call
F
Log2ratio
G
Number of probes in the aCGH call

Supplementary Figure S4
Detection of aCGH calls by WGS calls

A. Absolute counts of the true positive and false positive calls detected by each WGS callers with a 50% reciprocal similarity, for deletions (left) and gains (right). The horizontal line represents the total amount of true positive calls. Combining callers slightly increases the true positive deletions detection, but also the false positive ones. B. Two examples of deletion calls that are more precise in WGS than aCGH. In the left case, one wrongly labelled probe explains the marked difference in breakpoint localization. In the right case, this is due to the probe density. C. Scheme of the reciprocal similarity threshold requirement. The default threshold considered was 50% for each call (middle). Relaxing the threshold on the fraction of aCGH covered by WGS call increased the detection of calls, demonstrating the low precision of aCGH breakpoint localization. D. When lowering the fraction of the WGS call required to be covered by the aCGH call, more true but also false positive calls are detected.

-

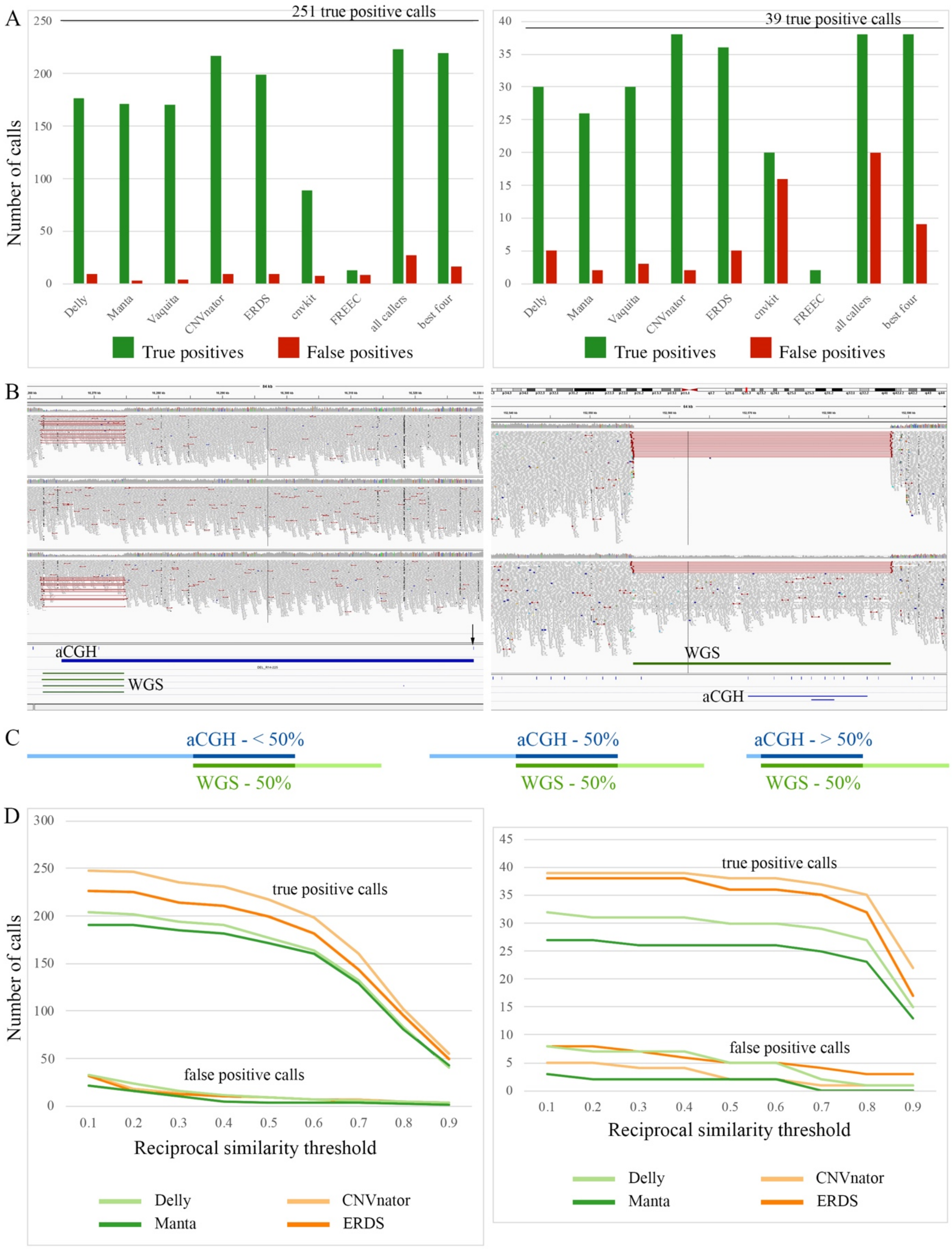
A
251 true positive calls
Number of calls
250
200
150
100
50
0
Delly
Manta
Vaquita
CNVnator
ERDS
cnvkit
FREEC
all callers
best four
True positives
False positives
39 true positive calls
40
35
30
25
20
15
10
5
0
B
aCGH
WGS
C
aCGH - < 50%
WGS - 50%
aCGH - 50%
WGS - 50%
aCGH - > 50%
WGS - 50%
D
300
true positive calls
false positive calls
0.1
0.2
0.3
0.4
0.5
0.6
0.7
0.8
0.9
Reciprocal similarity threshold
Delly
Manta
CNVnator
ERDS
45

Supplementary Figure S5
Overlap of callers

Repartition of overlap configurations per caller and type of call. Generally speaking, callers using the same signal show higher overlap than others. Deletions calls by ERDS are an exception and the most frequent category is called by all four callers, then both paired-end callers together with ERDS. Delly and CNVnator show high amounts of uniquely called deletions and gains, which can be put in relation with their lower fraction of supported calls.

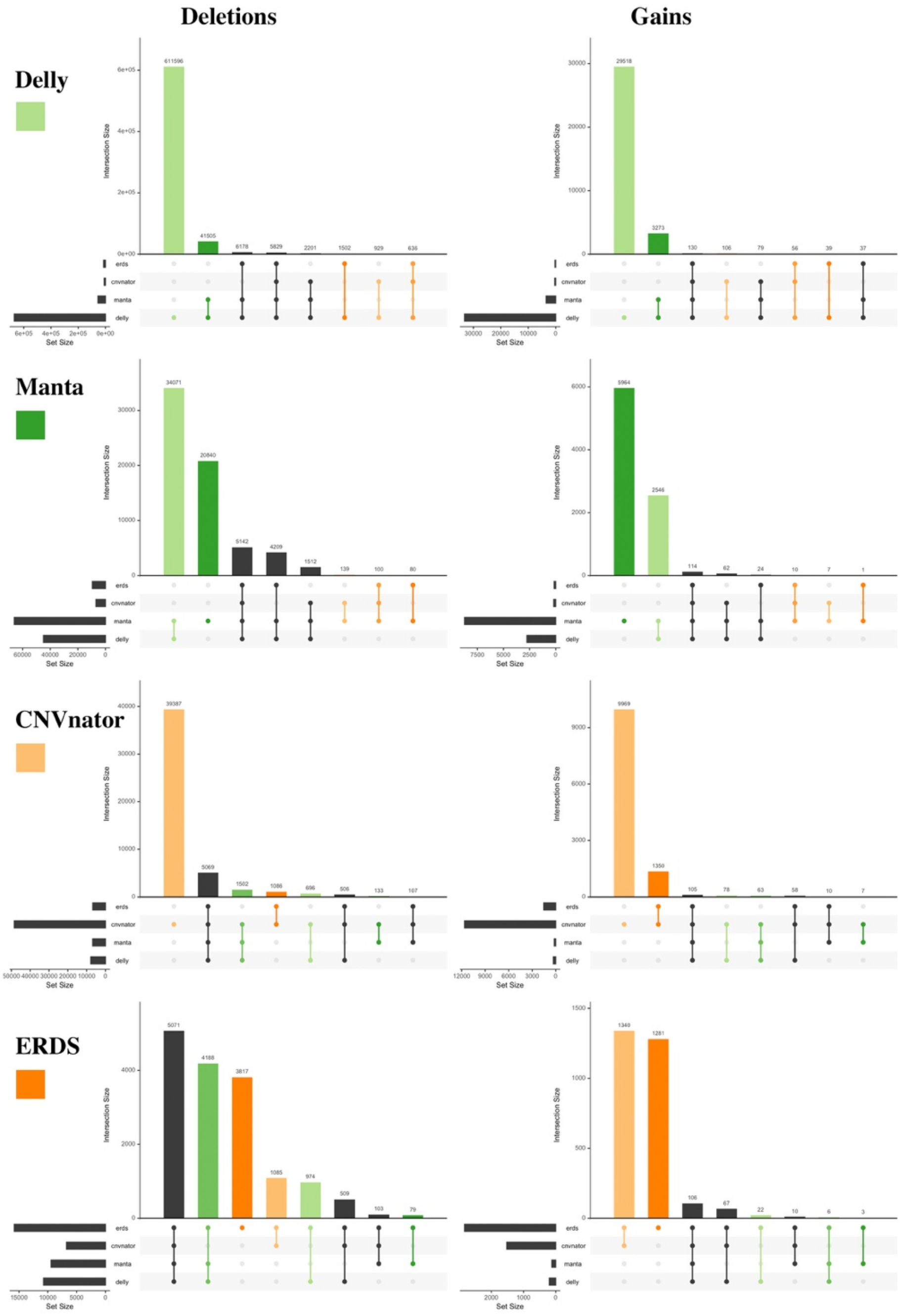

Supplementary Figure S6
Overlap of callers per inspection category

Repartition of overlap configurations per inspection category and type of call. With higher call certainty came more frequent overlaps. In both true positive deletions and gains, the most frequent category was calls detected by all four callers. In the false positive calls, unique callers and pairs are the most frequent. Bars are coloured when the call is covered by a unique caller.

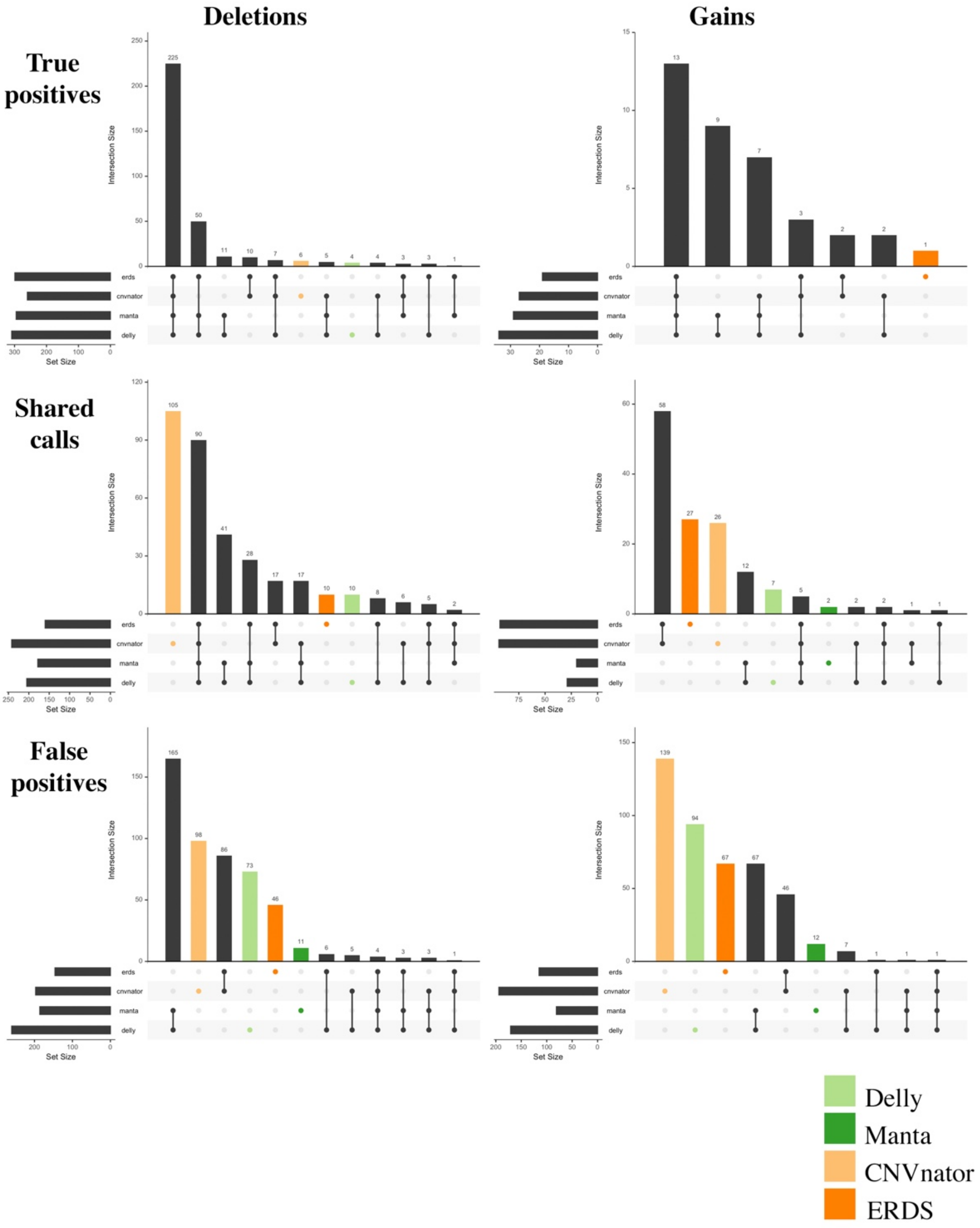

Supplementary Figure S7
Overlap of inspected WGS calls with mappability tracks

Calls were intersected with several mappability tracks: the DAC blacklisted regions (regions of the human genome with anomalous, unstructured, high read counts), Duke excluded regions (problematic regions for short sequence tag signal detection), a set of alternative scaffolds lifted over from hg38, and the Repeat Masker track (interspersed repeats and low complexity DNA sequences). A. False positives are not more frequently in DAC blacklisted regions. B. Deletions show a small enrichment for Duke excluded regions. C. This trend is more present for alternative scaffolds, where both deletions and gains are slightly enriched. D. There is no obvious difference in the Repeat Masker content of the calls categories, which is expected since repeats are located all over the genome, including in introns of coding genes.

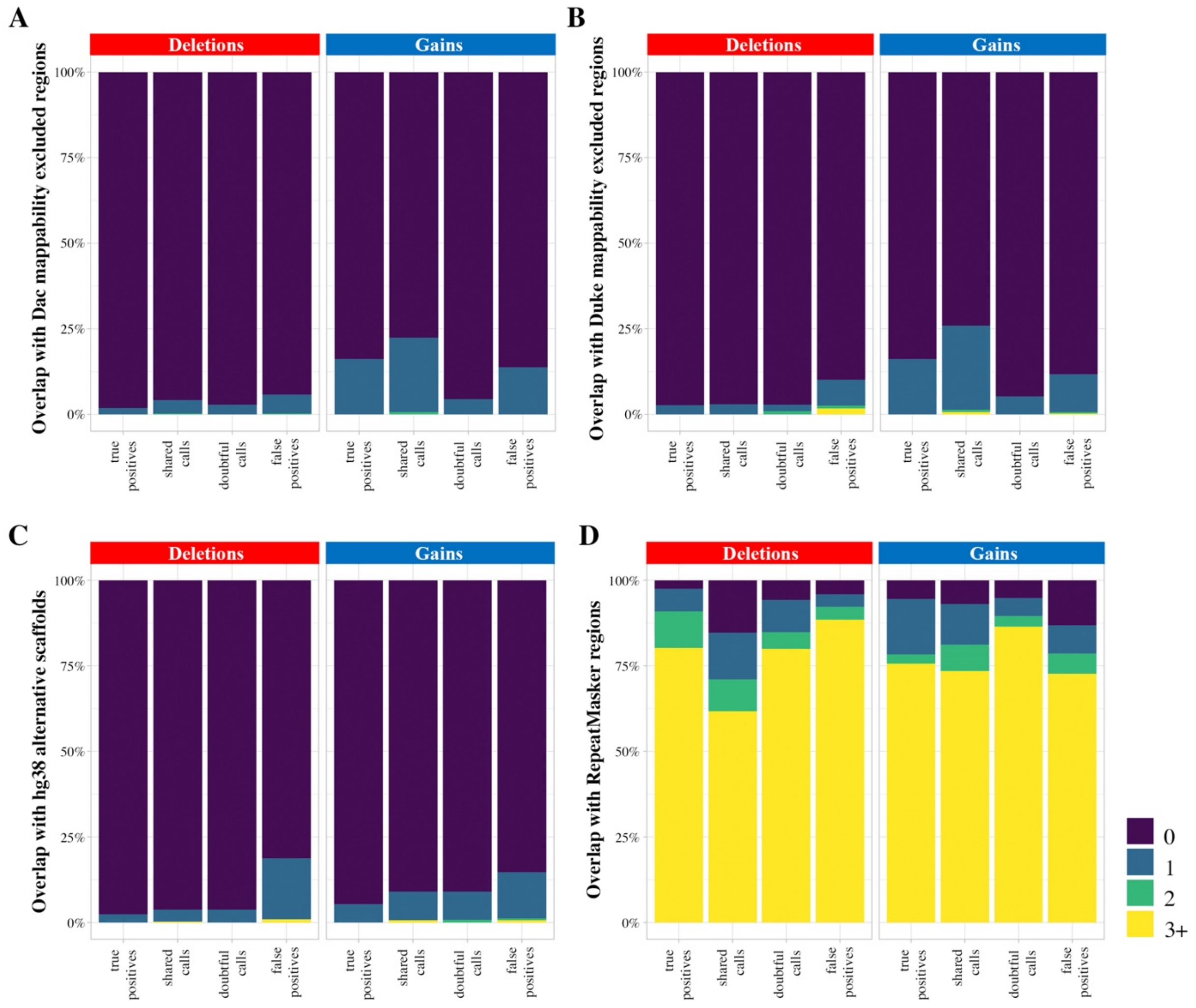

Supplementary Figure S8
Contingency values per caller and type of calls

For each caller and each filter described in the method section, several contingency values were calculated.
The sensitivity (Se) is the ability of the filter to detect true calls (fraction of true positive calls detected). The specificity (Sp) is the fraction of false positive calls that are indeed not detected. The predictive positive value (PPV) is the fraction of true positive calls in the total number of calls and was compared to the pre-filter PPV as a ratio (PPV ratio). The accuracy is the fraction of accurate judgments, *i.e.* the amount of true positive and true negative calls amongst all calls. "Intrinsic" refers to filtering with intrinsic calls properties (adjusted p-value for CNVnator, paired-end and split-read support fraction for Delly). "Delamp" stands for the removal of calls intersecting opposite calls with 75% reciprocal overlap (Supplementary Figure S9). Intersect 0.5 and 0.75 correspond to the intersection of similar caller type calls with respectively 50 and 75% of reciprocal overlap. Values are compared with the values that would be obtained without filtering ("no filter").

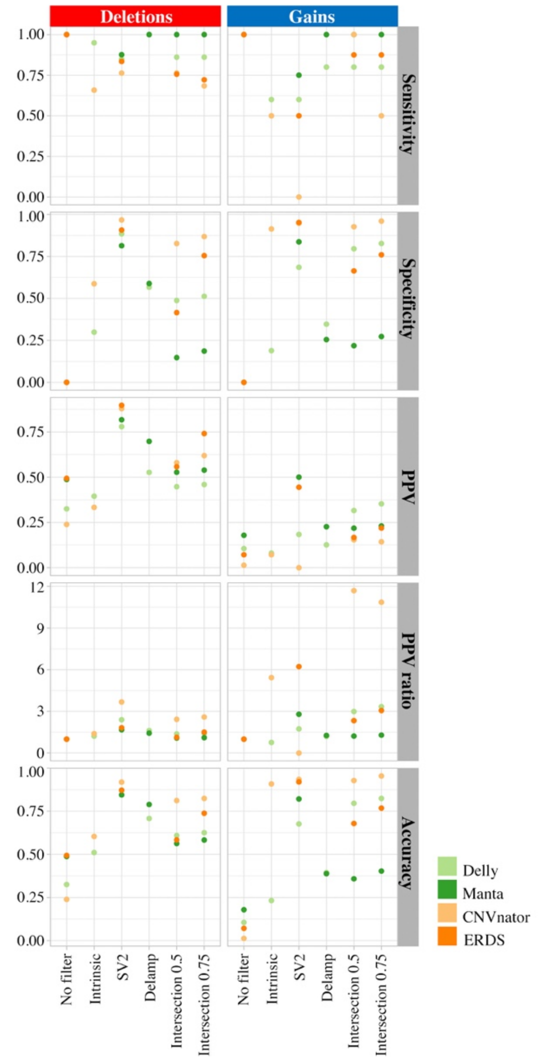

Supplementary Figure S9
False positive calls filtered out by the "delamp" filter

A. Proportion of deletions and gains that show an overlap with opposite call, in the Delly and Manta datasets. The problem is marginal for deletions but more prominent for gains, especially for Manta. B. Deletion-gain call at a threshold of 90%. The bottom track is the Repeat Masker track, showing occurrences of the MLT1A1 repeat at both ends of the call. It could then just be a mapping error with reads aligning to both occurrences of the repeated element. C. Deletion-gain call at a threshold of 75%, in a region flagged by many overlapping deletions and gains. D. Deletion-gain call at a threshold of 75%, which could doubtfully be a more complex event but is not supported by any coverage depth change.

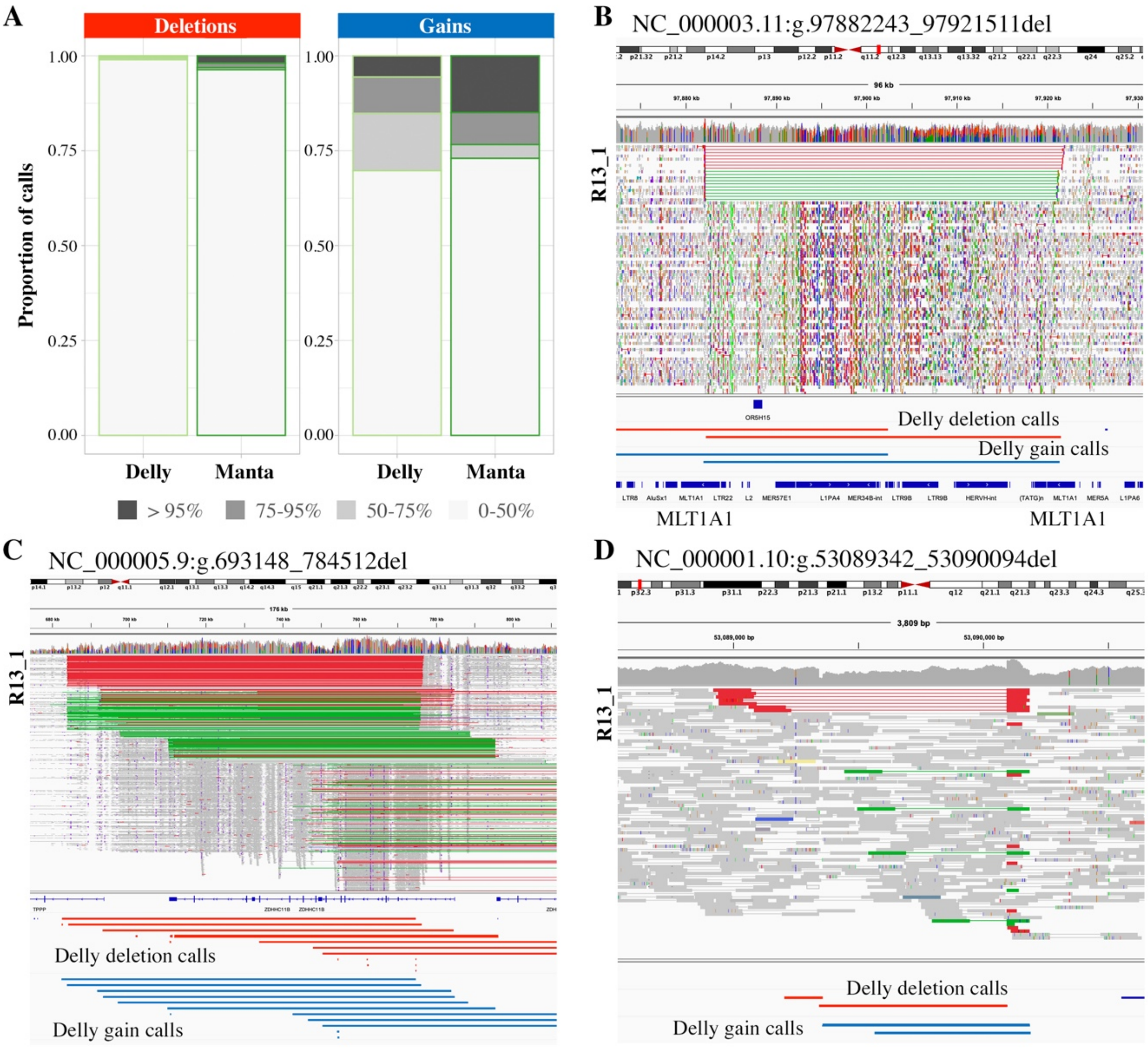

Supplementary Figure S10
Contingency values for combinations of tools

Sensibility, specificity (A), accuracy and positive predictive value (B) for single callers and combined approaches described in the Methods section. The positive and negative sets include all inspected calls from the four callers, which explains the always imperfect sensitivity. SV2 shows high specificity, but really low sensitivity for gains. The threshold of reciprocal overlap for intersections increases specificity while preserving sensitivity, hence 75% was deemed a good option.

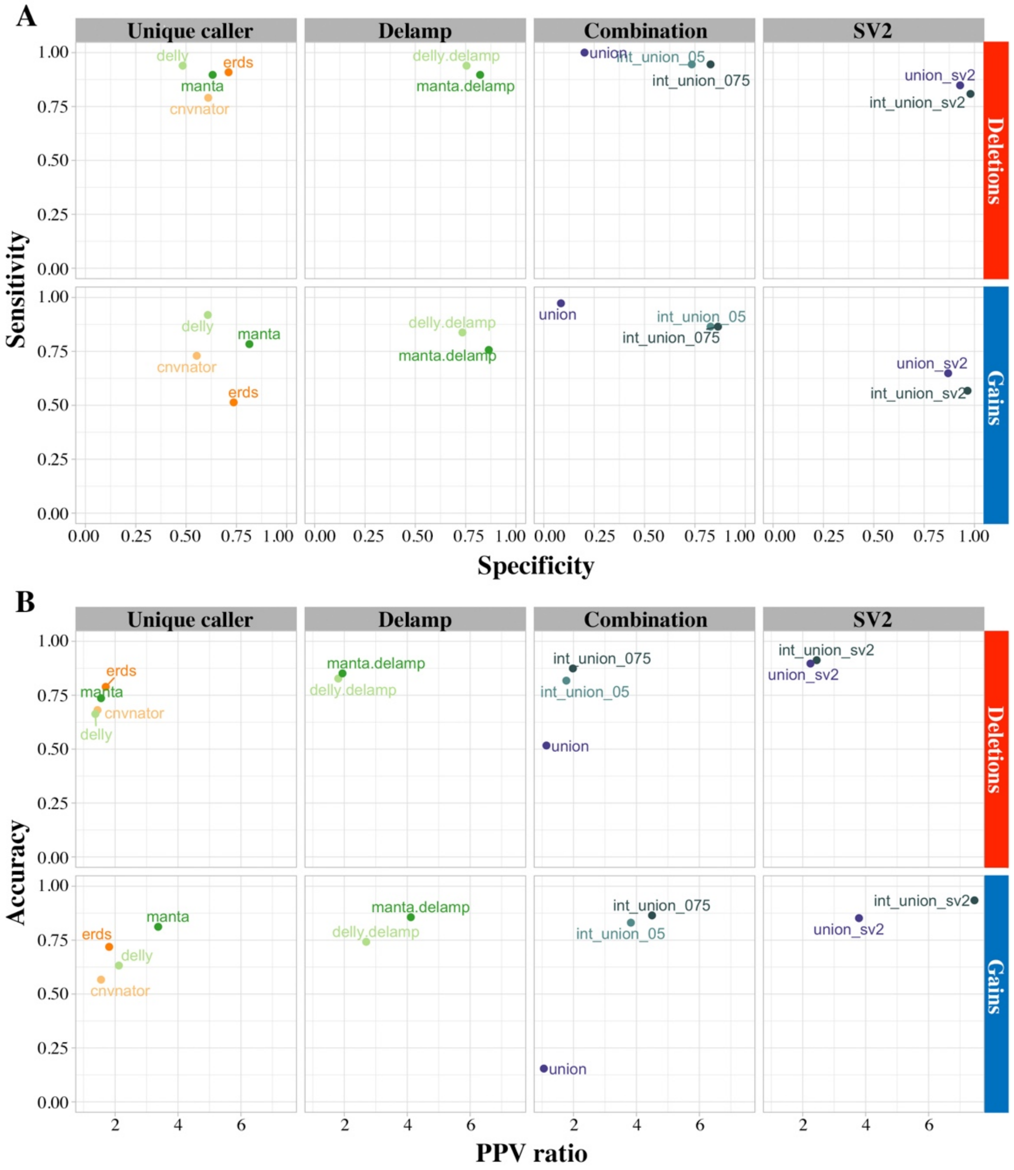

Supplementary Figure S11
Deletions from the intersection-union approach, not confirmed by qPCR

Despite showing clear support in IGV and being detected by all four callers, those two loci could not be confirmed by qPCR. They were found recurrently in the cohort, and overlapped with gnomAD SVs with frequency of 25.7% and 35.3%, hence they could be alternative loci.

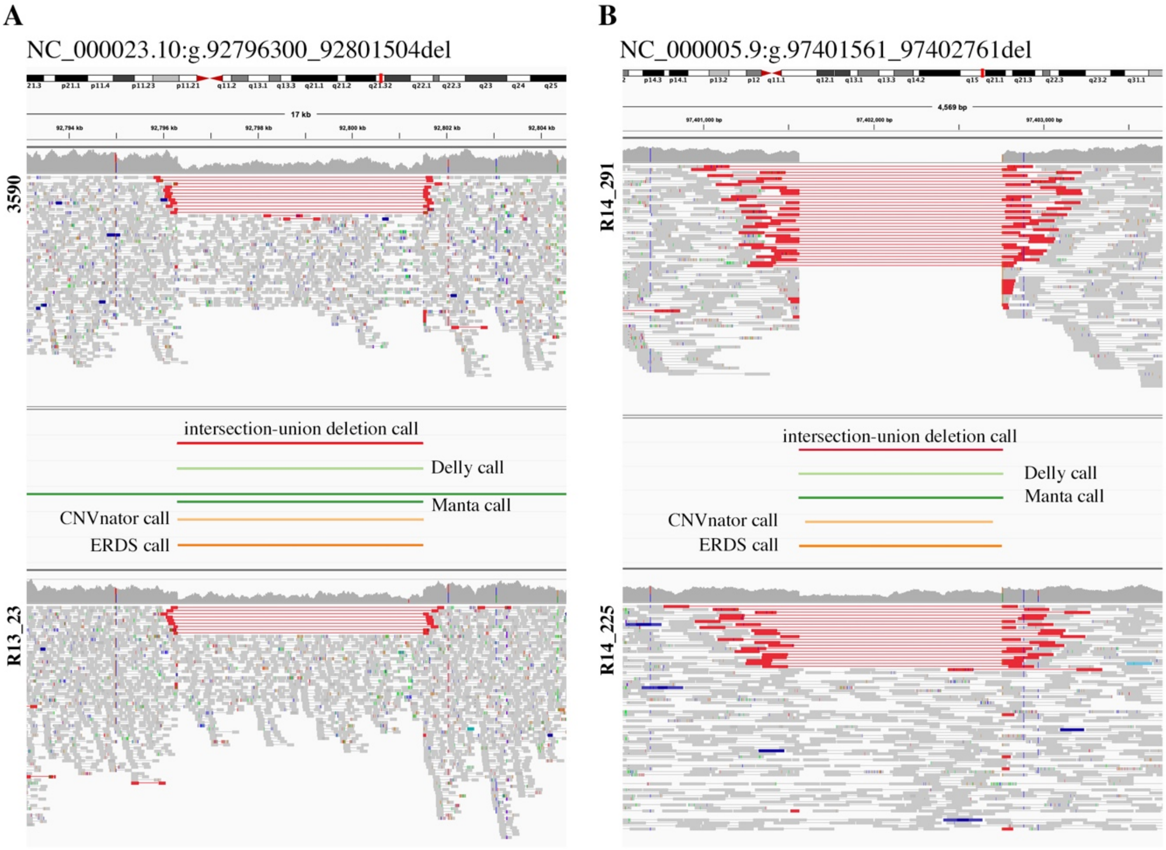

Supplementary Figure S12
ACGH probes in intersection-union calls

A. Number of aCGH probes in the intersection-union call. The calls yielded by the intersection-union approach are not only located in probes-devoid regions, as evidenced by the numbers of probes they contain, which increases with their size. B. Distribution of the amounts of probes in inspection categories. No obvious bias can be detected between the true and false positive calls.

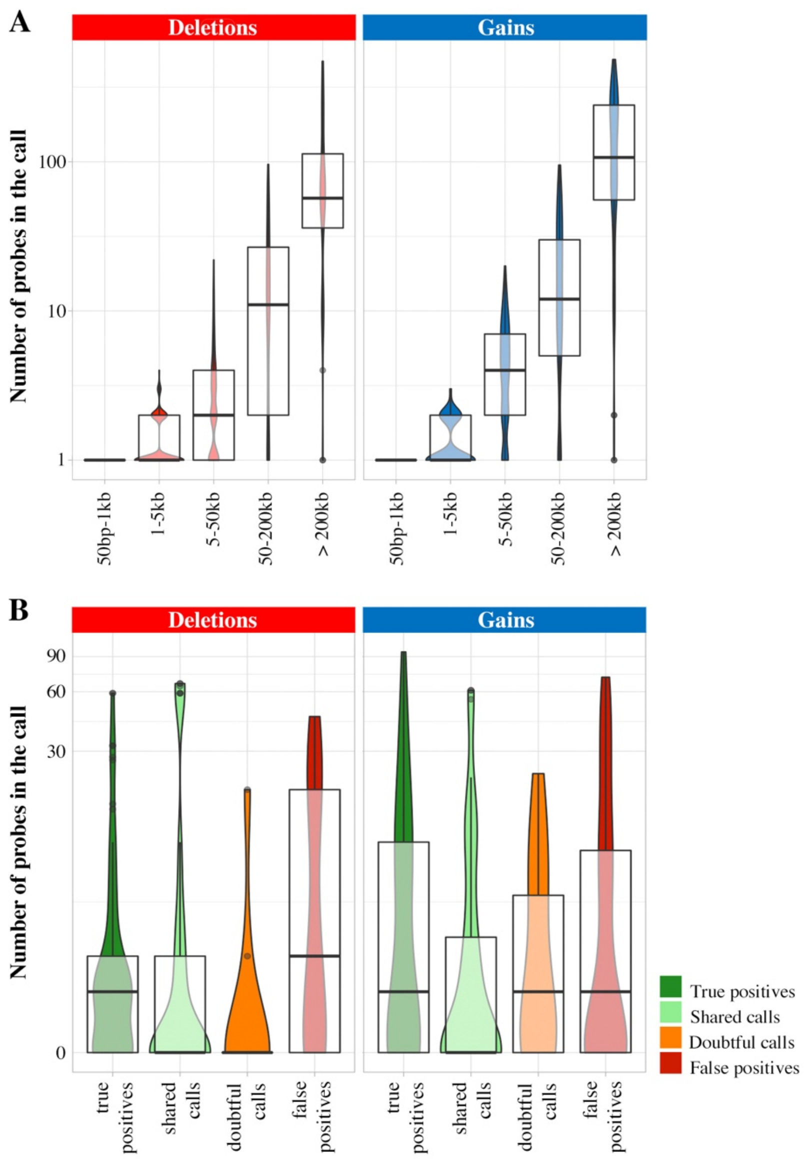

Supplementary Figure S13
Insertion call in patient 3430

In this patient, a gain was called by the intersection-union approach. It is detected by both coverage-based callers, and had previously been detected by aCGH, but characterized as VUS. Indeed, this patient with mirror-image polydactyly of the hands and feet showed no phenotypic overlap with a patient carrying a similar duplication. Coverage-based callers, like aCGH, were not sufficient to provide a molecular explanation to the phenotype. Having access to paired-reads, however, allowed to identify a fusion between intron 1 of *SHH* on chromosome 7, and intron 8 of *KDM4C* on the other hand, which could lead to *SHH* ectopic expression (Elsner, Mensah et al, in press).

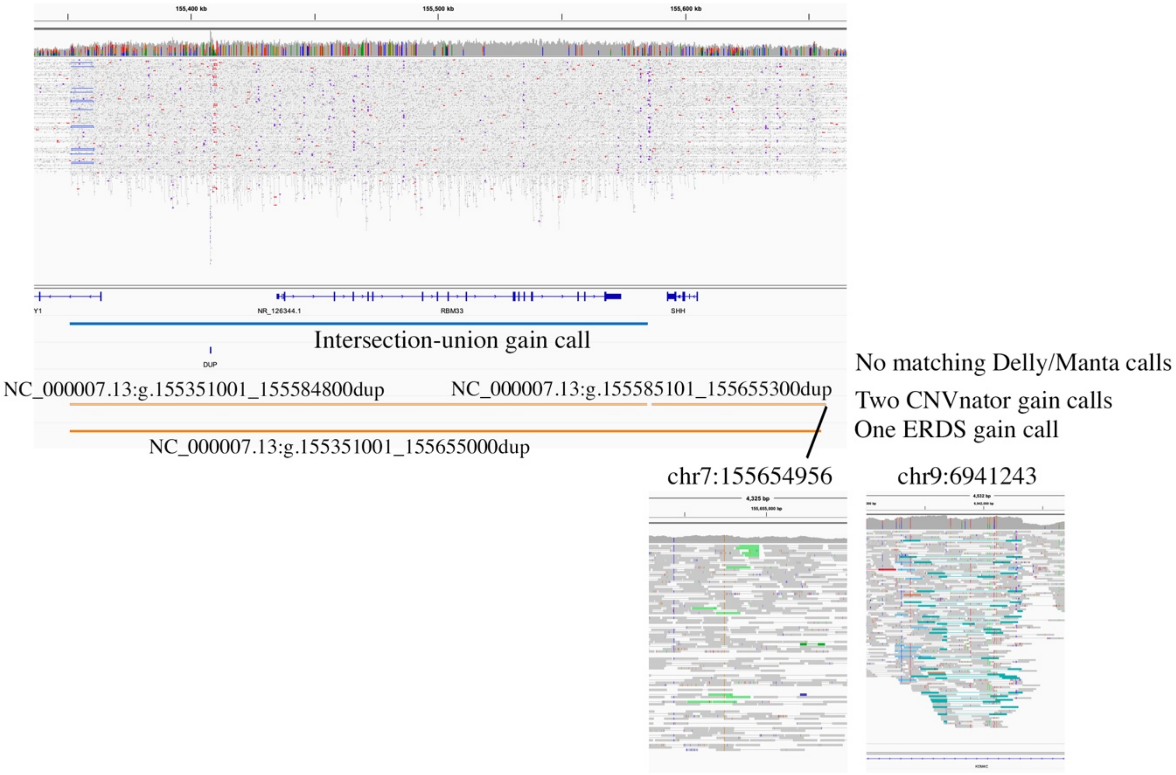

Supplementary Table S1
Average number of calls per caller and patient, for each size group and CNV type, for the ten patients of the training group.

Calls below 50 base pairs do not strictly fulfill the definition of CNV and were not considered in the total of counts.

**Deletions**	*<50kb*	***50bp-1kb***	***1-5kb***	***5-50kb***	***50-200kb***	***>200kb***	**Total**
cnvkit	0	0	18	144	43	42	247
**CNVnator**	0	**1830**	**1648**	**1082**	**242**	**47**	**4849**
**Delly**	34323	**65631**	**852**	**348**	**134**	**74**	**67039**
**ERDS**	0	**696**	**635**	**150**	**92**	**11**	**1584**
FREEC	0	0	449	2932	3182	2026	8589
**Manta**	0	**5545**	**575**	**249**	**71**	**170**	**6610**
Vaquita	26	8143	853	280	74	0	9350

**Gains**	*<50kb*	***50bp-1kb***	***1-5kb***	***5-50kb***	***50-200kb***	***>200kb***	**Total**
cnvkit	0	0	2	27	23	6	58
**CNVnator**	0	**3**	**476**	**603**	**75**	**8**	**1165**
**Delly**	0	**2760**	**219**	**159**	**120**	**65**	**3323**
**ERDS**	0	**29**	**40**	**119**	**74**	**22**	**284**
FREEC	0	0	1252	745	4	1	2002
**Manta**	7	**456**	**86**	**86**	**61**	**184**	**873**
Vaquita	0	196	130	113	73	0	512

Supplementary Table S2
Overlap between calls from different callers

Average counts of calls detected by each pair of two callers, versus average number of calls per caller. The overlap for deletions is in the bottom left half of the table, the overlap for gains in the upper right half. The coverage-based callers are highlighted in grey, while the paired-end based callers are left in white. Vaquita is a mixed caller and is highlighted in light grey.

	Average number of gains	1164	284	57	2003	3324	880	512
Average number of deletions		**CNVnator**	**ERDS**	**cnvkit**	**FREEC**	**Delly**	**Manta**	**Vaquita**
4849	**CNVnator**	-	152	15	5	30	19	25
1583	**ERDS**	677	-	14	3	26	13	17
247	**cnvkit**	118	43	-	0	4	3	3
8589	**FREEC**	34	4	20	-	15	4	3
101360	**Delly**	777	1415	33	23	-	352	399
6609	**Manta**	681	944	31	18	5694	-	190
9376	**Vaquita**	626	800	31	6	4705	2069	-

Supplementary Table S3
Detailed eyeballing results for the WGS calls of the training group

1278 deletions (585 from 1 to 5kb, 205 from 5 to 50kb, 488 from 50 to 200kb) and 748 gains (328 from 1 to 5kb, 420 from 5 to 50kb) from the four best callers (CNVnator, ERDS, Delly and Manta) were visually inspected in IGV as described in the methods section. True positive calls objectified by the presence of a coverage drop, paired-end abnormal signal or split-reads were separated in two categories: "true, 2-" when the signal was present in a maximum of two alleles, and "true, 3+" for supposed polymorphism with allele count above two in the trio. Calls were labeled as shared when the IGV profile appeared similar in the index and both parents, either heterozygous ("shared, het"), homozygous ("shared, hom"), or uncharacterized ("shared"). The true fraction (true calls among all calls) and supported fraction (true and shared calls among all calls) are reported. The total counts gathered for filters evaluation is indicated.

			Delly	Manta	CNVnator	ERDS	Total	Total true
Deletions	1-5kb	true, 2-	27	39	10	51	249	329
		true, 3+	31	32	17	42		
		shared, het	12	17	8	14		
		shared, hom	26	23	63	36		
		doubtful	15	7	15	22		**Total false**
		false	30	6	33	9	78	505
		true fraction	***0.41***	***0.57***	***0.18***	***0.53***		
		supported fraction	***0.68***	***0.90***	***0.67***	***0.82***		
		total counts	141	124	146	174		
	5-50kb	true, 2-	7	13	3	8	68	
		true, 3+	13	12	6	6		
		shared, het	1	7	2	4		
		shared, hom	11	17	7	4		
		doubtful	5	9	17	1		
		false	22	8	19	3	52	
		true fraction	***0.34***	***0.38***	***0.17***	***0.54***		
		supported fraction	***0.54***	***0.74***	***0.33***	***0.85***		
		total counts	59	66	54	26		
	50-200kb	true, 2-	1	1	0	3	12	
		true, 3+	0	0	2	5		
		shared, het	1	2	0	1		
		shared, hom	8	7	54	14		
		doubtful	8	5	0	1		
		false	112	88	69	106	375	
		true fraction	***0.01***	***0.01***	***0.02***	***0.06***		
		supported fraction	***0.08***	***0.10***	***0.45***	***0.18***		
		total counts	130	103	125	130		
							Total	**Total true**
Gains	1-5kb	true, 2-	8	9	1	1	19	37
		shared	12	10	13	31		
		doubtful	22	22	5	1		**Total false**
		false	84	16	60	33	193	435
		true fraction	***0.06***	***0.16***	***0.01***	***0.02***		
		supported fraction	***0.16***	***0.33***	***0.18***	***0.48***		
		total counts	126	57	79	66		
	5-50kb	true, 2-	7	3	1	7	18	
		shared	2	3	20	52		
		doubtful	22	46	10	5		
		false	43	39	90	70	242	
		true fraction	***0.09***	***0.03***	***0.01***	***0.05***		
		supported fraction	***0.12***	***0.07***	***0.17***	***0.44***		
		total counts	74	91	121	134		
	50-200kb	true, 2-	0	0	1	4		
		shared	0	1	7	7		
		doubtful	0	0	6	4		
		false	50	47	36	35		
		true fraction	0	0	0.02	0.08		
		supported fraction	0.00	0.02	0.16	0.22		
		total counts	50	48	50	50		

Supplementary Table S4
Contingency values for the filters used for each caller

For each caller and each filter described in the method section, several contingency values were calculated.
The sensitivity (Se) is the ability of the filter to detect true calls (fraction of true positive calls detected). The specificity (Sp) is the fraction of false positive calls that are indeed not detected. The positive predictive value (PPV) is the fraction of true positive calls in the total number of calls and was compared to the pre-filter PPV as a ratio (PPV ratio). The accuracy is the fraction of accurate judgments, *i.e.* the amount of true positive and true negative calls amongst all calls. "Intrinsic" refers to filtering with intrinsic calls properties (adjusted p-value for CNVnator, paired-end and split-read support fraction for Delly). "Delamp" stands for the removal of calls intersecting opposite calls with 75% reciprocal overlap (Supplementary Figure S9). Intersect 0.5 and 0.75 correspond to the intersection of similar caller type calls with respectively 50 and 75% of reciprocal overlap. Values are compared with the values that would be obtained without filtering ("no filter").

Delly

		No filter	*Intrinsic*	*SV2*	*Delamp*	*Intersect 0.5*	*Intersect 0.75*
Deletions	*Se*	1.00	0.95	0.85	1.00	0.86	0.86
	Sp	0.00	0.30	0.88	0.57	0.49	0.51
	PPV	0.33	0.39	0.78	0.53	0.45	0.46
	PPV ratio	1.00	1.21	2.40	1.62	1.38	1.41
	Accuracy	0.33	0.51	0.87	0.71	0.61	0.63
Gains	*Se*	1.00	0.60	0.60	0.80	0.80	0.80
	Sp	0.00	0.19	0.69	0.35	0.80	0.83
	PPV	0.11	0.08	0.18	0.13	0.32	0.35
	PPV ratio	1.00	0.76	1.74	1.20	2.99	3.34
	Accuracy	0.11	0.23	0.68	0.39	0.80	0.82

Manta

		No filter	*SV2*	*Delamp*	*Intersect 0.5*	*Intersect 0.75*
Deletions	*Se*	1.00	0.88	1.00	1.00	1.00
	Sp	0.00	0.81	0.59	0.15	0.19
	PPV	0.49	0.82	0.70	0.53	0.54
	PPV ratio	1.00	1.68	1.43	1.08	1.11
	Accuracy	0.49	0.84	0.79	0.56	0.58
Gains	*Se*	1.00	0.75	1.00	1.00	1.00
	Sp	0.00	0.84	0.25	0.22	0.27
	PPV	0.18	0.50	0.23	0.22	0.23
	PPV ratio	1.00	2.79	1.26	1.22	1.29
	Accuracy	0.18	0.82	0.39	0.36	0.40

CNVnator

		No filter	*Intrinsic*	*SV2*	*Intersect 0.5*	*Intersect 0.75*
Deletions	*Se*	1.00	0.66	0.76	0.76	0.68
	Sp	0.00	0.59	0.97	0.83	0.87
	PPV	0.24	0.33	0.88	0.58	0.62
	PPV ratio	1.00	1.39	3.68	2.43	2.59
	Accuracy	0.24	0.60	0.92	0.81	0.82
Gains	*Se*	1.00	0.50	0.00	1.00	0.50
	Sp	0.00	0.91	0.95	0.93	0.96
	PPV	0.01	0.07	0.00	0.15	0.14
	PPV ratio	1.00	5.43	0.00	11.69	10.86
	Accuracy	0.01	0.91	0.93	0.93	0.95

ERDS

		No filter	*SV2*	*Intersect 0.5*	*Intersect 0.75*
Deletions	*Se*	1.00	0.83	0.76	0.72
	Sp	0.00	0.91	0.42	0.75
	PPV	0.49	0.90	0.56	0.74
	PPV ratio	1.00	1.82	1.13	1.50
	Accuracy	0.49	0.87	0.58	0.74
Gains	*Se*	1.00	0.50	0.88	0.88
	Sp	0.00	0.95	0.66	0.76
	PPV	0.07	0.44	0.17	0.22
	PPV ratio	1.00	6.22	2.33	3.06
	Accuracy	0.07	0.92	0.68	0.77

Supplementary Table S5
Contingency values for pipeline options

For single callers and pipeline steps as described in the methods section, several contingency values were calculated. "Delamp" refers to the removal of calls designed as both gains and deletions by a single caller. Sensitivity (Se), specificity (Sp), positive predictive value (PPV), ratio to positive predictive value without filters (PPV ratio) and accuracy are reported, as described in Supplementary Table S4.

			Unique callers				Delamp		Combinations of callers			SV2	
		No filter	*Delly*	*Manta*	*CNVnator*	*ERDS*	*Delly*	*Manta*	*Union*	*Intersection-union 0.5*	*Intersection-union 0.75*	*Union + SV2*	*Intersection-union 0.75 + SV2*
**Deletions**	*Se*	1.00	0.94	0.90	0.79	0.91	0.94	0.90	1.00	0.95	0.95	0.85	0.81
	Sp	0.00	0.48	0.63	0.61	0.71	0.75	0.82	0.20	0.73	0.83	0.93	0.98
	PPV	0.39	0.54	0.61	0.57	0.67	0.71	0.77	0.45	0.70	0.78	0.89	0.96
	PPV ratio	1.00	1.37	1.55	1.44	1.70	1.81	1.94	1.14	1.77	1.98	2.25	2.44
	Accuracy	0.39	0.66	0.74	0.68	0.79	0.83	0.85	0.52	0.82	0.87	0.90	0.91
**Gains**	*Se*	1.00	0.92	0.78	0.73	0.51	0.84	0.76	0.97	0.86	0.86	0.65	0.57
	Sp	0.00	0.61	0.81	0.55	0.74	0.73	0.86	0.08	0.83	0.86	0.87	0.97
	PPV	0.08	0.17	0.26	0.12	0.14	0.21	0.32	0.08	0.30	0.35	0.30	0.58
	PPV ratio	1.00	2.12	3.37	1.55	1.81	2.70	4.11	1.06	3.82	4.50	3.79	7.46
	Accuracy	0.08	0.63	0.81	0.57	0.72	0.74	0.86	0.15	0.83	0.86	0.85	0.93

Supplementary Table S6
Number of calls per size per suggested pipeline

Average number of calls per patient in the training group, for each suggested pipeline approach, per size category. The repartition of calls per patient is plotted in Figure 4B.

		50bp-1kb	1-5kb	5-50kb	50-200kb	>200kb
**Deletions**	*Intersection-union 0.75*	3624.8	787.7	321.5	48.6	7.2
	Union + SV2	1174.2	2386.4	1089.8	48	6
**Gain**	*Intersection-union 0.75*	76.6	37.3	89.8	28.1	9.8

Supplementary Table S7
Detailed eyeballing results for the intersection-union calls

200 deletions (68 from 1 to 5kb, 32 from 5 to 50kb, 100 from 50 to 200kb) and 200 gains (33 from 1 to 5kb, 67 from 5 to 50kb, 100 from 50 to 200kb), and all variants above 200kb (72 deletions, 98 gains) from the intersection-union approach in the training patients were visually inspected in IGV as described in the methods section and the legend of Supplementary Table S3. Fractions of true and supported calls are notably higher than for single callers but decrease with increased size of the calls. For calls above 200kb especially, the true fraction remains extremely low. Number in parentheses indicate unique calls.

		**1-5kb**	**5-50kb**	**50-200kb**	**>200kb**
**Deletions**	true, 2-	36	11	14	0
	true, 3+	15	10	6	0
	shared, het	5	4	31	0
	shared, hom	8	2	7	0
	doubtful	1	0	9	3 (2)
	false	3	5	33	69 (36)
	true fraction	0.75	0.66	0.20	0.00
	supported fraction	0.94	0.84	0.58	0.00
	total counts	68	32	100	72 (38)
**Gains**	true, 2-	9	12	12	2 (2)
	true, 3+	0	0	0	3 (1)
	shared	13	23	18	10 (1)
	doubtful	2	3	8	4 (4)
	false	9	29	62	79 (32)
	true fraction	0.27	0.18	0.12	0.05
	supported fraction	0.67	0.52	0.30	0.19
	total counts	33	67	100	98

Supplementary Table S8
Calls from the intersection-union approach validated via qPCR

4 gains from 1.4 to 24.3kb, and 11 deletions from 1 to 109kb, all absent from the aCGH call set, were orthogonally checked by qPCR. All gains were validated, as well as 9/11 deletions. Similar signal was present in other patients for both deletions that were not confirmed. All coordinates refer to the hg19 genomic reference sequence. NA: not applicable

Type of CNV	Size	HGVS	Patient	Zygosity in IGV	qPCR result	Recurrent in cohort
Gain	24332	NC_000007.13:g.89229635_89253966dup	3590	NA	7q21.13 gain confirmed	No
Gain	12000	NC_000006.11:g.69230001_69242000dup	3590	NA	6q12.6 gain confirmed	No
Gain	6690	NC_000017.10:g.57524922_57531611dup	R14_291	NA	17q22 gain confirmed	No
Gain	1464	NC_000019.9:g.55466569_55468032dup	R14_291	NA	19q13.42 gain confirmed	No
Deletion	109000	NC_000004.11:g.70123401_70232400del	3590	Heterozygous	4q13.2 deletion confirmed	No
Deletion	9734	NC_000004.11:g.10392434_10402167del	R14_291	Heterozygous	4p16.1 deletion confirmed	Yes
Deletion	5205	NC_000023.10:g.92796300_92801504del	3590	Heterozygous	Xq21.32 deletion not confirmed	Yes
Deletion	4637	NC_000001.10:g.26460133_26464769del	3590	Heterozygous	1p36.11 deletion confirmed	No
Deletion	2552	NC_000010.10:g.84127814_84130365del	3590	Heterozygous	10q23.1 deletion confirmed	Yes
Deletion	2329	NC_000018.9:g.63766874_63769202del	3590	Heterozygous	18q22.1 deletion confirmed	Yes
Deletion	1758	NC_000023.10:g.32987322_32989079del	3590	Heterozygous	Xp21.1 deletion confirmed	Yes
Deletion	1673	NC_000020.10:g.1389143_1390815del	3590	Heterozygous	20p13 deletion confirmed	Yes
Deletion	1233	NC_000008.10:g.143397460_143398692del	3590	Heterozygous	8q24.3 deletion confirmed	No
Deletion	1201	NC_000005.9:g.97401561_97402761del	R14_291	Homozygous	5q15 deletion not confirmed	Yes
Deletion	1087	NC_000022.10:g.23478491_23479577del	R14_291	Homozygous	22q11.23 deletion confirmed	No

Supplementary Table S9
Quantification of intersection-union calls in regions targeted by aCGH

Calls issued from the intersection-union approach for the training patients were intersected with windows of respectively 1 and 10kb around the coordinates of aCGH probes. On average, more than 250 deletions and 50 gains above 5kb are detected in close proximity to those probes, hence the additional calls detected by aCGH are not limited to regions absent from the aCGH design.

		50bp-1kb	*1-5kb*	*5-50kb*	*50-200kb*	*> 200kb*
**Deletions**	*1kb interval*	1343.3	497.4	240.8	29.7	5.3
	10kb interval	3405.3	741.2	287.1	35.2	5.5
**Gains**	*1kb interval*	34.4	18.5	43.4	19.6	9
	10kb interval	69.8	25.5	55	20.7	9

Supplementary Table S10
Number of calls per pipeline for selected frequencies and regions sets

Average number of calls, for the training patients, for each suggested pipeline approach, compared to each caller alone. Filters were applied on the maximal frequency of overlapping calls in the gnomAD database, and on selected region sets. The list of genes implicated in limb malformation is available at the end of the supplementary data. Increasing the stringency of the frequency filter has a limited effect on reducing the call set size.

			Unique callers				Pipeline suggestions	
	Frequency filter	***Region filter***	*Delly*	*Manta*	*CNVnator*	*ERDS*	*Intersection-union 0.75*	*Union + SV2*
**Deletions**	-	none	101360	6609	4849	1583	4813	4704
	5%	none	97943	4270	4175	806	2180	2710
		UCSC exons	1827	233	846	106	110	403
		limb TADs	19438	865	685	172	450	469
	1%	none	97495	4135	4109	755	2041	2560
	0.1%	none	96889	4049	4045	724	1964	2445
		UCSC exons	1757	207	831	100	99	389
		limb TADs	19231	826	668	158	414	433
		UCSC exons + limb genes	65	34	37	2	2	22
**Gains**	-	none	3324	880	1164	284	242	-
	5%	none	3146	721	1069	260	188	-
		UCSC exons	298	175	385	132	67	-
		limb TADs	796	141	129	36	29	-
	1%	none	3062	694	1051	253	177	-
	0.1%	none	2865	665	1036	249	166	-
		UCSC exons	259	154	371	126	61	-
		limb TADs	716	132	120	33	25	-
		UCSC exons + limb genes	11	42	3	1	1	-

Supplementary Table S11
Number of aCGH calls per patient

Census of aCGH counts, per size, for each patient from the training group.

	**Deletions**			
	1-5kb	*5-50kb*	*50-200kb*	*> 200kb*
**3586**	4	17	2	0
**3590**	6	28	5	4
**R13_1**	2	35	5	3
**R13_23**	12	43	10	3
**R14_225**	9	27	11	1
**R14_291**	9	30	7	1
**R15_27**	4	23	6	3
**R15_66**	7	22	5	0
**R16_144**	8	28	7	2
**R16_30**	8	18	7	2
	**Gains**			
	1-5kb	*5-50kb*	*50-200kb*	*> 200kb*
**3586**	0	3	1	1
**3590**	2	12	5	3
**R13_1**	4	14	4	1
**R13_23**	0	17	3	1
**R14_225**	2	8	7	3
**R14_291**	2	11	3	1
**R15_27**	2	14	8	2
**R15_66**	1	9	4	2
**R16_144**	0	10	3	2
**R16_30**	0	12	6	1

Supplementary Table S12
Detailed inspection results for the aCGH calls of the training group

422 deletions and 184 gains called with aCGH were visualized from WGS data in IGV. True positive calls objectified by the presence of a coverage drop, paired-end (PE) abnormal signal or split-reads were separated in "true, 2-" when the signal was present in at most two alleles, and "true, 3+" for supposed polymorphism with allele count above two in the trio. Calls were labeled as shared when the IGV profile appeared similar in the index and both parents, either heterozygous ("shared, het"), homozygous ("shared, hom"), or uncharacterized ("shared, alt"). All calls above 200kb appeared as false positives, opposite calls or doubtful calls.

		Deletions	Gains
< 200kb	true, 2-	152	39
	true, 3+	97	0
	shared, het	11	2
	shared, hom	38	0
	shared, alt	0	2
	false	79	60
	opposite	7	55
	doubtful	22	12
> 200kb	false	13	10
	opposite	1	0
	doubtful	2	4
	**total**	**422**	**184**

Supplementary Table S13
Intersection of aCGH calls with gnomAD SVs, for each category after inspection in IGV.

Counts are based on intersections that reciprocally overlap with 50% of the SV length. Results are reported for identical type of calls as well as opposite types of calls (*e.g.* overlapping gains with deletions).

			Number of calls	Number of intersecting gnomAD SVs	Percentage of intersecting gnomAD SVs	Number of opposite gnomAD SVs	Percentage of opposite gnomAD SVs
**Deletions**	**True positives**	true, 2-	152	123	80.92	12	7.89
		true, 3+	97	75	77.32	0	0
	**Shared**	shared, het	11	7	63.64	0	0
		shared, hom	38	25	65.79	0	0
	**False positives**	false	79	5	6.33	4	5.06
		false, > 200kb	13	2	15.38	0	0
		opposite	7	0	0	1	14.29
		opposite, > 200kb	1	0	0	0	0
	**Doubtful**	doubtful	22	14	63.64	2	9.09
		doubtful, > 200kb	2	0	0	0	0
**Gains**	**True positives**	true, 2-	39	33	84.62	7	17.95
	**Shared**	shared, het	2	1	50	1	50
		shared, alt	2	1	50	0	0
	**False positives**	false	60	3	5	33	55
		false, > 200kb	10	0	0	0	0
		opposite	55	0	0	47	85.45
	**Doubtful**	doubtful	12	6	50	5	41.67
		doubtful, > 200kb	4	1	25	0	0

Supplementary Table S14
Detailed aCGH detection counts for each caller

Absolute number of calls detected by each caller, combination of all callers or the four best ones, for each of the aCGH eyeballing category as detailed in Supplementary Table S12. The coverage-based callers are highlighted in grey, while the PE callers are left in white. Vaquita is a mixed caller and is highlighted in light grey. Overall, the best two coverage-based callers perform better than paired-end callers, which could be explained because they look at the same signal type than aCGH.

		**True positives**		**Shared**			**Doubtful**	**False positives**	
		true, 2-	true, 3+	shared, het	shared, hom	shared, alt	doubtful	false	opposite
**Deletions**	*Delly*	116	61	7	22	0	4	9	0
	Manta	113	58	7	20	0	2	3	0
	Vaquita	112	58	7	22	0	3	4	0
	CNVnator	136	81	11	27	0	15	9	0
	ERDS	125	74	8	24	0	12	9	0
	cnvkit	46	43	4	16	0	9	6	1
	FREEC	7	6	1	1	0	3	7	1
	all callers combined	139	84	11	31	0	18	25	2
	Delly-Manta-CNVnator-ERDS	139	81	11	27	0	17	16	0
	array-CGH count	**153**	**98**	**11**	**38**	**0**	**24**	**92**	**8**
**Gains**	*Delly*	30	0	1	0	0	0	5	0
	Manta	28	0	1	0	0	0	2	0
	Vaquita	30	0	1	0	0	0	3	0
	CNVnator	38	0	2	0	2	5	2	0
	ERDS	36	0	2	0	1	6	5	0
	cnvkit	20	0	1	0	1	3	11	5
	FREEC	2	0	0	0	0	0	0	0
	all callers combined	38	0	2	0	2	10	15	0
	Delly-Manta-CNVnator-ERDS	38	0	2	0	2	8	9	0
	array-CGH count	**39**	**0**	**2**	**0**	**2**	**16**	**70**	**55**

Supplementary Table S15
Validation results for the intersection-union approach

For the 14 patients not used for the initial comparison, we visually inspected aCGH calls that had not been detected with the intersection-union approach and classified them as previously described (Supplementary Table S12). The calls labeled “better in WGS” were not considered as detected when intersecting the WGS calls with the aCGH calls with a reciprocal overlap of 50%; however, upon visual inspection, WGS proved to better characterize those calls.

	Deletions	Gains
**True positive**	33	4
**Shared**	4	5
**Doubtful**	1	3
**Better in WGS**	88	5
**False positive**	112	103
**Opposite**	7	90
**Total**	245	210

Supplementary Table S16
Census and intersection of calls sets for the NA12878 reference sample

The reference set of SVs for reference individual NA12878 was downloaded from ftp://ftp.1000genomes.ebi.ac.uk/vol1/ftp/phase3/integrated_sv_map/. Its aligned, downsampled to 30X, short-read genome was obtained on the Genome in a Bottle github page (https://github.com/genome-in-a-bottle/giab_data_indexes), and the Manta calls were extracted from the Manta original paper (PMID 26647377). Delly, CNVnator and ERDS were ran as described in the Methods section. The total number of calls for each caller, as for the intersection-union approach are reported, together with their overlap with the gold-standard call set for NA12878. The sensitivity is the fraction of positive calls that were detected, and is detailed for deletions in and out of repeats (ALU, LINE1, SVA) regions, as established in the reference dataset. The positive predictive value is the amount of calls that are indeed true positives and is calculated assuming that the gold-standard includes all true positive calls, hence possibly underestimated.

			Delly	*Manta*	*CNVnator*	*ERDS*	*Intersection-union 0.75*	*NA12878 set*
**Total number of calls**	**Deletions**	*All*	9561	4244	2670	1040	3131	
**Number of calls in NA12878 true positives set**		*Non repeats*	1093	1000	471	624	972	1310
		Repeats	644	574	48	14	551	672
**Sensitivity**		*Non repeats*	0.83	0.76	0.36	0.48	**0.74**	
		Repeats	0.96	0.85	0.07	0.02	**0.82**	
**Positive predictive value**		*All*	0.18	0.37	0.19	0.61	**0.49**	
**Total number of calls**	**Gains**	*All*	4072	419	1760	396	181	
**Number of calls in NA12878 true positives set**			3	2	6	5	6	8
**Sensitivity**			0.38	0.25	0.75	0.63	**0.75**	
**Positive predictive value**			0.00	0.00	0.00	0.01	**0.03**	

Supplementary Table S17
Contingency values of calls sets for the NA12878 reference sample, per size

Sensitivities and positive predictive values for each caller and the intersection-union approach are calculated as described in Supplementary Table S16, and reported per call type and size range. The paired-end based callers perform the best to detect small deletions, the coverage-based ones for larger calls or amplifications. Manta and ERDS show higher positive predictive values, indicating more reliable calls.

				Delly	*Manta*	*CNVnator*	*ERDS*	*Intersection-union 0.75*	*Number of calls in NA12878 set*
**Sensitivity**	**Deletions**	*50bp-1kb*	*Non repeats*	0.79	0.81	0.11	0.17	**0.70**	699
			Repeats	0.96	0.85	0.07	0.02	**0.82**	666
		1-5kb	*Non repeats*	0.93	0.75	0.57	0.84	**0.81**	431
		5-50kb	*Non repeats*	0.81	0.64	0.82	0.78	**0.75**	167
			Repeats	1.00	1.00	0.17	0.17	**1.00**	6
		50-200kb	*Non repeats*	0.54	0.46	0.77	0.92	**0.77**	13
	**Gains**	*5-50kb*	*All*	0.38	0.25	0.75	0.63	**0.75**	8
**Positive predictive value**	**Deletions**	*50bp-1kb*	*All*	0.21	0.32	0.26	0.58	**0.50**	
		1-5kb		0.29	0.68	0.22	0.65	**0.51**	
		5-50kb		0.10	0.59	0.19	0.81	**0.40**	
		50-200kb		0.02	0.24	0.04	0.13	**0.26**	
	**Gains**	*5-50kb*	*All*	0.00	0.05	0.01	0.03	**0.08**	

Supplementary Data – list of genes implicated in limb malformations

A2ML1, ABCA12, ABCC8, ABCC9, ACAN, ACOX1, ACTB, ACTG2, ACVR1, ADAMTS10, AGA, AGL, AGPAT2, AGTR1, AHI1, AIP, AKT1, AKT3, ALDOA, ALOX12B, ALPL, ALX4, ANKH, ANKRD11, ANO5, ANTXR1, ARHGAP31, ARID1A, ARID1B, ARL13B, ARL4D, ARL6, ARVCF, ASXL1, ATF2, ATN1, ATP1A3, ATP6V0A2, ATP7A, ATPAF2, ATR, ATRIP, ATRX, AXIN1, B3GALTL, B4GALT7, B9D1, B9D2, BANF1, BBIP1, BBS1, BBS10, BBS12, BBS2, BBS4, BBS5, BBS7, BBS9, BCL2L11, BCLAF1, BCOR, BHLHA9, BLM, BLOC1S3, BMF, BMP15, BMP2, BMP4, BMPER, BMPR1B, BRAF, BRCA2, BRF1, BRIP1, BTK, BTRC, BUB1, BUB1B, BUB3, C12ORF57, C5orf42, CA2, CANT1, CASR, CBS, CC2D2A, CCDC8, CCND2, CD40LG, CD96, CDH3, CDKL5, CDKN1C, CDX4, CENPE, CENPJ, CEP120, CEP152, CEP290, CEP41, CEP57, CHCHD10, CHD7, CHN1, CHRNA1, CHRND, CHRNG, CHST14, CHST3, CLCF1, CLCN5, CLCN7, CLEC3B, COL10A1, COL11A1, COL11A2, COL12A1, COL1A1, COL2A1, COL3A1, COL5A1, COL5A2, COL6A1, COL6A2, COL6A3, COL7A1, COL9A1, COL9A2, COL9A3, COMP, COMT, COX4I2, COX7B, CPSF1, CREBBP, CRLF1, CSF1R, CSPP1, CTC1, CTDP1, CTSC, CTSD, CTSK, CUL4B, CUL7, CYP19A1, D2HGDH, DACT1, DCLRE1C, DCR, DDB2, DEAF1, DHCR7, DHODH, DKC1, DKK1, DLAT, DLL3, DLX3, DLX5, DLX6, DMP1, DNM2, DOCK6, DOK7, DOLK, DPAGT1, DPH1, DPPA4, DRG2, DSPP, DUSP3, DYM, DYNC2H1, EBP, ECE1, ECEL1, EDA, EDAR, EDN3, EDNRB, EFEMP2, EFNB1, EFTUD2, EIF2AK3, EIF2B1, EIF2B2, EIF2B3, EMG1, ENPP1, EOGT, EPHX1, ERCC1, ERCC2, ERCC4, ERCC5, ERCC6, ERF, ERRFI1, ESCO2, ETV4, EVC, EVC2, EVX1, EVX2, EXT1, EXT2, EZH2, FAM134B, FAM134C, FAM58A, FAM63A, FAM83H, FANCA, FANCB, FANCC, FANCD2, FANCE, FANCF, FANCG, FANCI, FANCL, FANCM, FAS, FBLN5, FBN1, FBN2, FBXW4, FCER1G, FERMT1, FGD1, FGF16, FGF18, FGF8, FGFR1, FGFR2, FGFR3, FGFRL1, FKBP10, FKBP8, FLNA, FLNB, FMR1, FOS, FOXG1, FOXH1, FRAS1, FREM1, FREM2, FSHR, FTO, FTSJ1, FURIN, G6PC3, GAD1, GALNT3, GATA1, GATA4, GATA6, GBA, GDF1, GDF5, GDNF, GGT1, GHR, GJA1, GJB2, GJB3, GJB4, GJB6, GLI2, GLI3, GNAI2, GNAS, GNAS-AS1, GNB4, GNE, GNPTAB, GNPTG, GP1BB, GPC3, GPC6, GPR101, GRIP1, GUSB, H19, HAPLN1, HBB, HCK, HDAC2, HDAC4, HDAC6, HDAC8, HES7, HESX1, HEXA, HHAT, HIC1, HIRA, HMGA2, HOXA10, HOXA11, HOXA13, HOXA2, HOXA3, HOXA4, HOXA5, HOXA6, HOXA9, HOXD12, HOXD13, HPGD, HRAS, HSD17B10, HSPB1, HSPG2, HUWE1, HYAL1, HYLS1, IBSP, IDS, IDUA, IFITM5, IFT122, IFT140, IFT172, IFT27, IFT43, IFT80, IFT88, IGBP1, IGF1, IGF1R, IGF2, IGFALS, IGHMBP2, IHH, IKBKG, INF2, INPP5E, INPPL1, INS, INSR, IRAK3, IRF5, IRF6, IRX5, ITGA10, ITGA2B, ITGA3, JAG1, JAG2, JDP2, KAT2A, KAT6B, KCNH1, KCNJ1, KCNJ11, KCNQ1OT1, KCTD10, KDF1, KDM5C, KDM6A, KIAA0196, KIAA0586, KIAA1279, KIAA1715, KIF1A, KIF22, KIF7, KL, KLB, KLLN, KMT2D, KRAS, KREMEN1, KRT10, KRT14, KRT17, L1CAM, LAMA5, LAMB3, LBR, LBX1, LEMD3, LFNG, LIFR, LIG4, LMBR1, LMNA, LMX1B, LONP1, LPIN2, LRP2, LRP4, LRP5, LRPPRC, LTBP2, LTBP3, LZTFL1, MAFB, MAP2K1, MAP2K2, MASP1, MATN3, MBD4, MBD5, MBTPS2, MECOM, MECP2, MED12, MEF2C, MEGF8, MEOX1, MESP2, MFAP5, MFN2, MGP, MIB1, MKKS, MKS1, MMACHC, MMP13, MMP9, MPZ, MRPS16, MUSK, MVK, MYBPC1, MYH3, MYH8, NAA10, NAGLU, NBR1, NEU1, NFATC1, NFKB2, NGFR, NHP2, NHS, NIPBL, NKX2-5, NKX2-6, NLRP3, NOD2, NOG, NOTCH1, NOTCH2, NOTCH3, NOV, NPHP1, NPPC, NPR2, NR5A1, NRAS, NRTN, NSD1, NSUN2, NTNG1, OAS1, OBSL1, OCRL, OFD1, ORC4, OSR2, PAK3, PALB2, PAX3, PCNT, PCYT1A, PDE4D, PDE6D, PDGFRB, PDHA1, PDS5B, PEPD, PEX10, PEX5, PEX7, PHC1, PHEX, PHF6, PHF8, PHGDH, PHOSPHO1, PHYH, PIEZO2, PIGO, PIGV, PIK3CA, PIK3R2, PITX1, PKD1, PLCG2, PLEC, PLK4, PLOD1, PLOD2, PNPLA6, POR, PORCN, POSTN, POU1F1, PPP5C, PPT1, PQBP1, PRKAR1A, PRLR, PSMC3IP, PTCH1, PTCH2, PTEN, PTH1R, PTHLH, PTK7, PTPN11, PTPN6, PTPN9, PUF60, PVRL1, RAB23, RAB3GAP1, RAB3GAP2, RAD21, RAD51C, RAF1, RAG1, RAG2, RAI1, RAPSN,

RARA, RASA2, RB1, RBBP8, RBM10, RBM8A, RBPJ, RDH10, RECK, RECQL4, RELA, RET, RIN2, RIPK4, RIPPLY2, RIT1, RMRP, RNF216, ROR2, RPGRIP1, RPGRIP1L, RPL26, RPS6KA3, RPS7, RUNX2, RXRA, RYK, SALL1, SALL4, SATB2, SBDS, SC5DL, SCARF2, SCN9A, SCX, SDCCAG8, SDHB, SDHC, SDHD, SEMA3A, SEMA3E, SETD2, SF3B4, SFN, SH3PXD2B, SHFM1, SHH, SHOC2, SIM1, SIX3, SKI, SLC16A2, SLC17A5, SLC26A2, SLC29A3, SLC34A1, SLC35A3, SLC35D1, SLC37A4, SLC39A13, SLC39A4, SLC52A2, SLC6A8, SLC9A6, SLCO2A1, SLX4, SMAD3, SMARCA4, SMARCAD1, SMARCB1, SMARCE1, SMC1A, SMC3, SMO, SMOC1, SMPD3, SMS, SNAP25, SNAP29, SNRPB, SNX10, SOS1, SOST, SOX11, SOX5, SOX9, SRCAP, SSR4, STAMBP, STAT3, STK11, STK3, STK4, STX16, SUFU, SUMF1, SYK, SYT2, TAP1, TAP2, TBC1D32, TBCE, TBX1, TBX15, TBX2, TBX22, TBX4, TBX5, TBXAS1, TCF12, TCIRG1, TCOF1, TCTN1, TCTN2, TFAP2A, TFAP2B, TGDS, TGFB1, TGFB2, TGFB3, TGFBR1, TGFBR2, THBS3, TM7SF2, TMEM107, TMEM138, TMEM216, TMEM231, TMEM237, TMEM67, TMEM70, TNFRSF11A, TNFRSF11B, TNFRSF1A, TNFSF11, TNNI2, TNNT3, TP63, TPM2, TRAF3IP1, TRAF6, TRAPPC2, TREX1, TRIM32, TRIM37, TRIP11, TRPC3, TRPS1, TRPV4, TSC1, TSC2, TSHB, TTC21B, TTC8, TUBB3, TWIST1, TWIST2, TXNL4A, UBA1, UBE2A, UBE3A, UFD1L, UPF3B, VCAN, VCP, VDR, VHL, VPS13B, WAS, WDR19, WDR34, WDR35, WDR60, WDR73, WDR81, WFIKKN1, WHSC1, WISP3, WNK1, WNT10B, WNT4, WNT5B, WNT7A, WRN, XYLT1, ZC4H2, ZDHHC9, ZFPM2, ZIC1, ZIC4, ZMPSTE24, ZNF141, ZNF423, ZNF469
